# Multilingualism at the Market: A Pre-registered Immersive Virtual Reality Study of Bilingual Language Switching

**DOI:** 10.5334/joc.359

**Published:** 2024-04-17

**Authors:** Alex Titus, David Peeters

**Affiliations:** 1 Radboud University, Centre for Language Studies, Nijmegen, the Netherlands; 2 Max Planck Institute for Psycholinguistics, Nijmegen, the Netherlands; 3 Tilburg University, Department of Communication and Cognition, TiCC, Tilburg, the Netherlands

**Keywords:** bilingual language production, language switching, virtual reality, ecological validity

## Abstract

Bilinguals, by definition, are capable of expressing themselves in more than one language. But which cognitive mechanisms allow them to switch from one language to another? Previous experimental research using the cued language-switching paradigm supports theoretical models that assume that both transient, reactive and sustained, proactive inhibitory mechanisms underlie bilinguals’ capacity to flexibly and efficiently control which language they use. Here we used immersive virtual reality to test the extent to which these inhibitory mechanisms may be active when unbalanced Dutch-English bilinguals i) produce full sentences rather than individual words, ii) to a life-size addressee rather than only into a microphone, iii) using a message that is relevant to that addressee rather than communicatively irrelevant, iv) in a rich visual environment rather than in front of a computer screen. We observed a reversed language dominance paired with switch costs for the L2 but not for the L1 when participants were stand owners in a virtual marketplace and informed their monolingual customers in full sentences about the price of their fruits and vegetables. These findings strongly suggest that the subtle balance between the application of reactive and proactive inhibitory mechanisms that support bilingual language control may be different in the everyday life of a bilingual compared to in the (traditional) psycholinguistic laboratory.

## Introduction

A large part of the world’s population regularly communicates in more than one language (Grosjean, 1989). One example can be found in most European universities, where a multitude of languages are spoken in a variety of contexts. A student may, for instance, converse with another student in English while having just concluded a meeting with a supervisor in Dutch. How do bilinguals use and transition between their languages with relative ease? The answer is not trivial, especially in light of clear evidence indicating that bilinguals do not have multiple language networks to switch on and off, but rather make use of an integrated lexical system where elements from both languages are typically activated in parallel and constantly compete for selection (e.g., Colomé, 2001; De Groot, 2011; Dijkstra, 2005; Dijkstra & van Heuven, 1998; Duyck et al., 2007; Hermans et al., 1998; Kroll et al., 2008; Kroll & Stewart, 1994; Moon & Jiang, 2012; Poulisse & Bongaerts, 1994).

A common way to experimentally study how bilinguals manage and control their different languages while speaking has been to collect data using a cued language-switching paradigm. In this computer task, bilinguals typically name pictures or digits in either their first language (L1) or their second language (L2) as a function of an often-arbitrary cue (e.g., a color cue) that indicates the language to respond with (e.g., Costa & Santesteban, 2004; Declerck & Philipp, 2015; MacNamara et al., 1968; Meuter & Allport, 1999). In a classic 2 × 2-design, speed and accuracy of single-word responses can be compared between switch trials (on which the language used differs from the previous trial) and non-switch trials (on which the language used is the same as on the previous trial) and between the bilingual’s dominant (often: first-learned) and non-dominant (often: second) language (Meuter & Allport, 1999).

This cued language-switching paradigm has yielded at least three relatively robust and theoretically interesting findings. First, switching languages typically takes longer compared to not switching languages (e.g., Bobb & Wodniecka, 2013; Kleinman & Gollan, 2016; Meuter and Allport, 1999). This *switch cost* is canonically interpreted in terms of the *reactive inhibition* of task schemas and/or individual translation equivalents (e.g., Green, 1998; Green & Abutalebi, 2013; Monsell, 2003). Indeed, the influential Inhibitory Control Model suggests that bilinguals via task schemas exert inhibitory control over a non-target language in order to effectively activate and select a target language (Green, 1998). It is also sometimes assumed that the lexical items corresponding to the trial-irrelevant language may be temporarily inhibited (e.g., Peeters et al., 2014). Specifically, once a cue indicates that a different language is to be used on trial *n* compared to trial *n–1*, bilinguals need to inhibit the active task schema ‘Name in Language A’ and activate the competing and temporarily inhibited task schema ‘Name in Language B’. Overcoming the inhibition of the previously suppressed task schema arguably takes some time and effort that is not required on non-switch trials.

Second, several studies using the cued language-switching paradigm have found that unbalanced bilinguals, defined as bilinguals that are substantially more proficient in one of their two languages, may counter-intuitively respond more quickly in their non-dominant L2 compared to their dominant (and often: first-learned) L1, which gives the perception of a temporarily *reversed language dominance* (e.g., Baus et al., 2015; Christoffels et al., 2007; Costa & Santesteban, 2004; Costa et al., 2006; Declerck et al., 2020; Declerck & Philipp, 2015; Gollan & Ferreira, 2009; Kleinman & Gollan, 2016; Kroll et al., 2008; Liu et al., 2019; Peeters et al., 2014; Peeters & Dijkstra, 2018; Verhoef et al., 2009; 2010). This finding is commonly interpreted to reflect bilinguals’ capacity of inhibiting their stronger L1 in a sustained way throughout a task. It is most commonly observed for unbalanced bilinguals with a relatively high second language proficiency, while studies testing bilinguals with a relatively low L2 proficiency do not consistently report it (cf. Calabria, et al., 2012; Declerck et al., 2013, Experiment 3; Filippi et al., 2014; Fink & Goldrick, 2015; Ivanova & Hernandez, 2021). The reversed language dominance effect, if observed, seems most evident in situations in which bilinguals need to accommodate the use of both their languages, such as when they are required to occasionally and unpredictably switch between languages for a prolonged period of time.

Indeed, the Adaptive Control Hypothesis proposes that, in dual-language contexts, a bilingual inhibitory control mechanism may act in a *proactive* manner (Green & Abutalebi, 2013). Such a dual-language context is indeed defined as a “context in which both languages are used but typically with different speakers” (Green and Abutalebi 2013, p. 518). The idea is that by suppressing the dominant language to a certain extent in a sustained way, language production in the non-dominant language becomes relatively easier throughout a task in which the involvement of both languages is required. Hence in addition to the trial-by-trial, reactive inhibitory process reflected in switch costs, reversed language dominance is commonly taken to reflect a proactive and *sustained inhibition of the L1*.

Third, the cued language-switching paradigm has often elicited so-called *mixing costs* when unbalanced bilinguals’ naming performance on single-language blocks is compared to non-switch trials during experimental blocks in which languages are mixed (e.g., Christoffels et al., 2007; Los, 1996; Peeters & Dijkstra, 2018; Prior & Gollan, 2011). This finding – that bilinguals name the same pictures or digits faster in a single-language context compared to the trials on which they do not have to switch in a mixed language context – can be taken to confirm that indeed *sustained inhibition* may be applied to the dominant language in mixed language environments, particularly when these mixing costs are indeed larger for the L1 than for the L2 (Christoffels et al., 2007; Peeters & Dijkstra, 2018; Prior & Gollan, 2011). When such an asymmetry in mixing costs is observed, switch costs are typically symmetrical (Christoffels et al., 2007; De Bruin et al., 2014; Declerck et al., 2013; Gollan & Ferreira, 2009; Mosca & Clahsen, 2016; Peeters, & Dijkstra, 2018; Wang et al., 2009). These findings hence suggest an interplay between reactive and sustained inhibitory mechanisms involved in bilingual language production.

In sum, unbalanced bilinguals seem to exert control over their languages using a combination of both reactive (trial-by-trial) and more sustained inhibitory mechanisms, as indicated by clear and relatively uncontroversial empirical findings (switch costs, reversed language dominance, mixing costs). In addition to the Inhibitory Control Model discussed above, a variety of theories of (aspects of) bilingual language production and control have been proposed to account for (some of) these observations (e.g., Branzi et al., 2014; Costa et al., 1999, 2006; Dylman & Barry, 2018; Finkbeiner et al., 2006; La Heij, 2005; Philipp et al., 2007; Poulisse & Bongaerts, 1994; Runnqvist et al., 2012, 2019). Irrespective of which of these theoretical models best explains the observed response time (RT) patterns, it remains an open question how relevant the behavior bilingual participants display in the lab is for everyday situations in which bilinguals switch from one language to another. Indeed, the relatively isolated and static laboratory settings in which these strict computer experiments take place are quite different from the visually rich and dynamic environments in which natural communication typically occurs. In everyday life, bilinguals do not switch as a function of artificial cues – they typically talk to actual addressees rather than into a microphone in a sound-proof booth, and they commonly produce utterances that consist of more than one word at a time (Heredia & Altarriba, 2001; Myers-Scotton, 2006). To what extent are the proposed inhibitory mechanisms involved in bilingual language switching as observed under strict experimental lab conditions also active under more natural circumstances? It seems clear that the study of human cognition should attempt to develop experimental paradigms that resemble the richness of the everyday environments in which our behavior typically takes place (e.g., Blanco-Elorrieta & Pylkkänen, 2018; Hamilton & Huth, 2018; Hari et al., 2015; Hasson et al., 2018; Peeters, 2019; Willems, 2015). After all, one hopes our theories of cognition will generalize to everyday situations.

Over the last decades, researchers have attempted to enhance the ecological validity of the cued language-switching paradigm in at least three ways. First, paradigms have been created that elicit free-choice, voluntary language switches (e.g., De Bruin et al., 2018; Gollan & Ferreira, 2009; Jevtović et al., 2020). Specifically, such studies often compare the RTs of cued switching against voluntary switching to examine lexical availability of each language. In the latter condition, participants are typically instructed to name a picture with the first word that comes to mind, regardless of the language that word belongs to. Compared to the traditional language-switching paradigm, the results from voluntary switching conditions have sometimes shown a *reduction* in switch costs or even *cost-free* switching between languages (Blanco-Elorrieta & Pylkkänen, 2017; De Bruin et al., 2018; Gollan & Ferreira, 2009; Gollan et al., 2014; Gross & Kaushanskaya, 2015; Jevtović et al., 2020; Kleinman & Gollan, 2016; Reverberi et al., 2018). In addition, in voluntary switching studies, sometimes “mixing benefits” have been observed in that RTs have been faster in mixed compared to single-language blocks (De Bruin et al., 2018; Jevtović et al., 2020). Across the board, these findings suggest that switching or mixing languages as a function of an artificial cue may require greater effort compared to (voluntary) language switching in day-to-day scenarios. Nevertheless, in everyday life, bilinguals may sometimes switch languages as a function of an external cue, such as when switching between different interlocutors (Peeters & Dijkstra, 2018).

Second, other studies have therefore enhanced the ecological validity of the cue that signals which language bilingual participants need to respond with (Blanco-Elorrieta & Pylkkänen, 2015; Hartsuiker, 2015; Liu et al., 2019; Martin et al., 2016; Molnar et al., 2015; Peeters, 2020; Peeters & Dijkstra, 2018; Smith et al., 2020; Timmer et al., 2017; 2019; Woumans et al., 2015; Zhang et al., 2015). An important cue to language in everyday life may be the face of a familiar interlocutor. It has indeed been observed that faces may prime a language, such that bilinguals are faster in producing words to a listener in a language that matches the presumed language identity of that listener (Woumans et al., 2015; cf. Blanco-Elorrieta & Pylkkänen, 2015; Molnar et al., 2015). Moreover, faces of listeners unknown to the speaker but with a clear presumed cultural identity, and even the presence of images of well-known iconic cultural artifacts (e.g., the Great Wall of China or the Statue of Liberty), may facilitate speaking in a language congruent with that face or image (Zhang et al., 2013; see also Roychoudhuri et al., 2016). In sum, the presence of a motivated language cue may steer bilinguals towards the use of one of their languages.

Third, a handful of studies have elicited language switches between utterances that consist of more than a single word. After all, speakers in natural situations typically produce more than one word per utterance. One study had Polish-English bilinguals describe actions in a sentence in one of their languages as a function of a cue and found substantial switch costs (Tarlowski et al., 2013). A study on German-English bilinguals however observed that switch costs may disappear when bilinguals switch languages while producing sentences that are syntactically similar and correct in both their languages (Declerck & Philipp, 2015; see also Gollan & Goldrick, 2016; 2018; Gullifer et al., 2013; Johns & Steuck, 2021). These mixed results, reminiscent of mixed result patterns in the domain of language switches in a sentence context in bilingual language comprehension (e.g., Ibáñez et al., 2010; Proverbio et al., 2004), suggest that more research is needed to test under what circumstances switch costs (and reversed language dominance and mixing costs for that matter) may occur or disappear.

In sum, lab-based studies strongly suggest that unbalanced bilinguals engage inhibitory control mechanisms when switching between their dominant and non-dominant languages when artificially cued to do so. Several studies have attempted to enhance the ecological validity of the cued language-switching paradigm by either inducing voluntary language switches, using motivated language cues such as faces, or eliciting switches between sentences rather than between isolated words. The results from these studies suggest that switch costs may reduce or, in certain cases, even disappear altogether. Potential modulations of the traditional reversed language dominance and mixing costs as a function of the presumed ecological validity of the experimental paradigm have received less attention. Here, we build upon previous work by taking the next step and have bilingual participants produce full-sentences in a rich and dynamic experimental environment in which their spoken message is communicatively relevant to their life-size addressee. In particular, we aim to gradually work towards a natural environment in the lab to further test the extent to which both reactive and sustained inhibitory mechanisms support bilingual language production in everyday life. We thereby hope that traditional studies and novel paradigms may go hand in hand in illuminating the cognitive processes supporting bilingual language switching capacities.

### The present study

The present study used immersive virtual reality to elicit language switches in unbalanced Dutch-English bilinguals in the lab. Compared to traditional experimental setups in this domain, such as the one used in the seminal study by Meuter and Allport (1999), we attempted to enhance the ecological validity of the paradigm by i) having bilinguals produce full sentences rather than individual words, ii) to a life-size addressee rather than only into a microphone, iii) using a message that is relevant to that addressee rather than irrelevant to that microphone, iv) in a rich everyday visual environment rather than in front of a computer screen. The main aim of the current study was therefore to test whether theories of bilingual language control, that have typically been based on findings obtained in computer experiments, generalize to some of the rich and dynamic everyday situations in which bilinguals typically switch between languages.

We note that the current experimental setup aimed to *move towards* a naturalistic experimental environment that resembles everyday bilingual language use. However, the paradigm clearly still lacked aspects of naturalistic bilingual communication, such as the limited spontaneity of the interaction between participant and virtual addressees, and the use of single sentences that are repetitive in their syntactic structure. Nevertheless, we opted for the current virtual reality approach as it allowed for immersing participants in a visually rich environment while maintaining the required experimental control to collect informative data in a reliable way (Peeters, 2019). Earlier work has indeed shown that participants may communicate to virtual agents in the way they would to human interlocutors (Heyselaar et al., 2017; Pan et al., 2016), particularly when researchers take into account any potential uncanny valley effects (Mori, 1970; Shin et al., 2019). Future research may build on the present study by including additional naturalistic factors (e.g., increased spontaneity of the dialog, a better balance between language production and comprehension) into the experimental mix.

The population of Dutch-English unbalanced bilinguals we investigate has yielded robust and consistent findings in previous studies that had participants from this population name pictures using single words. In a total of five experiments, symmetrical switch costs (i.e., switch costs of a similar magnitude for L1 versus for L2), reversed language dominance (i.e., faster RTs for L2 than for L1), and asymmetrical mixing costs (i.e., larger mixing costs for L1 than for L2) have been reliably observed (Peeters & Dijkstra, 2018; Peeters, 2020). No differences in these result patterns were found when a traditional picture naming computer setup was used compared to when participants performed the same language switching task in a virtual environment (Peeters & Dijkstra, 2018). Hence, we know that any potential novelty effects caused by having participants switch languages in virtual environments do not cause traditional findings to change (cf. Peeters, 2019; Tromp et al., 2018).

Below, we will introduce and report the results of two experiments. Experiment 1 served as a baseline computer experiment in which we tested whether simply having this population of unbalanced bilinguals produce sentences (rather than individual words) in a language switching task as a function of artificial color cues changed the commonly observed results. If reactive and sustained inhibitory mechanisms support bilinguals’ language switching capacities in situations where they express themselves in full sentences rather than isolated words, we would observe slower RTs for switch trials compared to non-switch trials (i.e., switch costs), and slower RTs for L1 trials compared to L2 trials (i.e., reversed language dominance) in a mixed-language block. In addition, we would see faster RTs for trials in single-language blocks compared to for non-switch trials in a mixed-language block, particularly for the L1 (i.e., asymmetrical mixing costs).

In Experiment 2, we tested a separate participant sample from the same bilingual population in an immersive virtual marketplace where they acted as the store owner of a fruit and vegetable stand. Similar to Experiment 1, participants made use of cues in order to respond in the context-appropriate language. However, instead of artificial color cues, participants encountered virtual agents with distinctive features (e.g., hair color, glasses, etc.) that they knew understood only one language (English or Dutch). If theories of bilingual language control generalize to everyday situations, we would also here observe switch costs, reversed language dominance, and asymmetrical mixing costs (cf. Bobb & Wodniecka, 2013; Costa & Santesteban, 2004; Declerck & Philipp, 2015; Christoffels et al., 2007; Kroll, et al., 2008; Peeters & Dijkstra, 2018; Peeters, 2020).

However, if the result patterns (switch costs, reversed language dominance, asymmetrical mixing costs) typically observed for this bilingual population were found only in Experiment 1, and not in Experiment 2, these findings would indicate that the presumed degree of naturalness of an experimental switching paradigm may indeed influence what result patterns are observed (cf. Blanco-Elorrieta & Pylkkänen, 2017; Gollan et al., 2014; Gollan & Ferreira, 2009; Gollan & Goldrick, 2018; Jevtović et al., 2020; Woumans et al., 2015). Such an observation would be taken to suggest that theoretical models of bilingual language control must explicitly take into account the linguistic, visual, and interactive context in which bilinguals switch between languages if they wish to explain cognitive mechanisms involved in everyday bilingual communication outside the lab.

## Experiment 1

### Method

#### Participants

Forty-eight L1 speakers of Dutch (*M* age = 22.8; age range: 18–30 years old; 39 female, 9 male) participated in this experiment. The chosen sample size was based on three criteria. First, according to recent recommendations (Brysbaert, 2019; Brysbaert & Stevens, 2018), a study with a 2 × 2 design that assumes sufficient statistical power (>90%), given a medium effect size (0.4), and intending to use a multiple regression for its analysis should test between 46 and 52 participants. Second, the earlier studies most similar to the current study (Peeters 2020; Peeters & Dijkstra, 2018) tested only half as many proposed participants *(N =* 24) for their experiments and were able to find robust and replicable effects. Third, an a priori G*Power (Faul et al., 2007) power analysis based on a 2 × 2 multiple regression analysis of variance suggested to test 42 participants to achieve enough statistical power (>90%) for a medium effect size (i.e., 0.4). We opted for testing the exact number of 48 participants as full counterbalancing of experimental trial lists required a sample size that was a multiple of 24 (see below). Participants were financially compensated for their time (10 euros per hour).

**Table 1:** Participant characteristics for Experiment 1: average score on the L2 English LexTALE test, average score on the *AX*-CPT test, self-reported age of acquisition (‘AoA’, in years of age) and proficiency (‘SRP’; based on a 1–7 Likert scale) with regards to listening, speaking, reading, and writing in both L1 Dutch and L2 English, and self-reported average hours of use per day of L2 English. For comparison with Table 6.


MEASURE	AVERAGE	SD

LexTALE	79.9	12.37

*AX*-CPT	–0.03	0.05

L1 Listening AoA	0.0	0.00

L1 Speaking AoA	0.5	0.90

L1 Reading AoA	1.7	2.39

L1 Writing AoA	1.9	2.65

L1 Listening SRP	7.0	0.00

L1 Speaking SRP	7.0	0.00

L1 Reading SRP	7.0	0.00

L1 Writing SRP	7.0	0.14

L2 Listening AoA	7.9	3.58

L2 Speaking AoA	9.4	3.25

L2 Reading AoA	10.0	2.23

L2 Writing AoA	10.2	2.59

L2 Listening SRP	6.1	0.85

L2 Speaking SRP	5.3	1.17

L2 Reading SRP	5.9	1.16

L2 Writing SRP	5.3	1.29

L2 Hours of use per day	5.6	3.73


#### Stimuli

Twenty colored images were created for this experiment. From the 20 total items (see Appendix A), 16 served as test items and four as practice items. In light of Experiment 2, the items are a mixture of fruits and vegetables commonly found at a marketplace stand, such as a pumpkin, peach, and orange (see Appendix A). The test items were chosen to have Dutch-English non-cognate names (e.g., English *potato*, Dutch *aardappel*). To allow for some variation in the to-be-produced sentences (see below), they were equally divided into four different cost sets (30, 50, 70, and 90 cents). Conversely, to facilitate task performance on practice trials that preceded the test blocks, practice items were chosen to have cognate names (e.g., Dutch *appel* vs. English *apple*) and all cost 80 cents. Furthermore, we assured that all 20 items were able to be purchased as singular items (e.g., tomato or cauliflower) and all had the same (common) gender in Dutch (i.e., *de* perzik, *de* tomaat; *the* peach, *the* tomato). We chose four color cues (blue, pink, purple, yellow) that were matched to each language, with two cues per language for each participant (i.e., 2:1 language-cue mapping; e.g., De Bruin et al., 2014; Heikoop et al., 2016; Jevtović et al., 2020; Peeters, 2020; Zheng et el., 2020), such that switching between languages was not confounded with switching between cues. The match between color cues and language was counterbalanced across participants and lists (see below).

#### Procedure

After providing informed consent, participants took part in an experiment that consisted of seven main stages: i) a price memorization block in which participants learned the price of each object presented in the experiment, ii) a first practice block (16 trials) aiming at practicing the item names in Language A (e.g., Dutch), iii) a baseline block (64 trials) in which participants produced full sentences in Language A (e.g., Dutch), iv) a second practice block (16 trials) in which participants practiced using the item names in Language B (e.g., English), v) a second baseline block (64 trials) in which participants produced full sentences in Language B (e.g., English), vi) a mixed block (256 trials) in which participants produced full sentences while both languages (50% L1 Dutch, 50% L2 English) were intermixed across trials (50% language switch trials, 50% language repeat trials), vii) a separate set of control tests that included the LexTALE English proficiency test (Lemhöfer & Broersma, 2012), the *AX*-CPT Baseline task (taken from Gonthier et al., 2016), and a language proficiency questionnaire (LHQ3, taken from Li et al., 2020).

Baseline blocks were hence always preceded by a practice block in the same language, and the mixed block always followed the baseline blocks. The order of presentation of single-language practice and baseline blocks (i.e., two Dutch blocks preceding two English blocks, or vice versa) was counterbalanced across participants. Below, we describe each stage of the experiment in further detail.

##### Price Memorization Block

**Figure 1:**
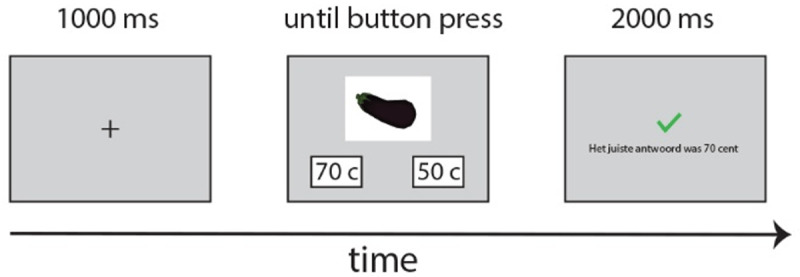
Example of one trial in the Price Memorization Block. This block familiarized participants with the prices of each of the items to facilitate sentence production in subsequent blocks.

The goal of the price memorization block was to familiarize participants with the prices of all objects. Items were divided into their five price sets (30, 50, 70, 80, and 90 cents). At the start of the block, participants reviewed a booklet with pictures of all 20 items, and the cost of each item (i.e., 30, 50, 70, 80, or 90 cents) for a total of five minutes. They then completed a 2 Alternative Forced Choice (2 AFC) task. An individual trial began with a fixation cross (1s), followed by the presentation of a picture (600 × 450 pixels) of an item slightly above the center of a computer screen and two possible prices at the bottom left and bottom right of the screen (see Figure 1). Participants were instructed to choose the correct response by pressing either the left or the right button on a button box. There was always one correct answer and one incorrect answer presented, the latter taken randomly from the prices used in the experiment. For example, if the correct answer was 50 cents and presented on the bottom left of the screen, on the right side of the screen one of the four other possibilities was presented (i.e., 30, 70, 80, or 90 cents) at random. The location on the screen of the correct and incorrect answers was counterbalanced (i.e., 50% of the correct answers appeared to the left bottom side of the monitor, 50% on the right bottom side). Items were presented in a random order.

Participants received feedback after their decision on each trial. If their response was correct, a green V appeared. If their response was incorrect, a red X appeared. Then, regardless of whether their response was correct, feedback appeared in Dutch, “Het juiste antwoord was Y cent” (“The correct answer was Y cents”) where Y denoted the correct price. Participants were instructed to respond as quickly and accurately as possible. All test and practice items were used and each item was presented a minimum of two times, leading to 40 trials to start with. Subsequently, participants needed to score above 80% correct after a minimum of 20 presented trials in order to move on to the Language A Practice Block. As such, this block consisted of at least 60 trials. If the participant did not reach at least 80% correct after a total of 120 trials, the block was repeated from the start. If they again did not reach 80% correct before trial 120, the experiment was stopped and the participant was replaced, which did not happen for any of the participants. After the final trial of this block, participants were given a small break until they felt comfortable to move to the Language A Practice Block.

##### Language A Practice Block: Single-Word Picture Naming and Color Cue Familiarization

The goal of this block was to familiarize participants with the images, their names, and the color cues that were used in baseline and mixed blocks. This practice block focused on one language only (i.e., Dutch or English, counterbalanced across participants). At the start of the block, participants briefly reviewed a booklet with pictures of all 20 items and their names in the respective language (Dutch or English) to-be-used in the block. Subsequently, on a computer screen, all 16 target images were presented once, one at a time, in random order of presentation. Participants were instructed to name each item in either their L1 Dutch or their L2 English. Target items were presented as pictures in the center of a computer screen. Additionally, one of the two color cues that were associated with the relevant language for a participant were presented above the picture to help familiarize them with the relation between color cue and language for the subsequent Baseline and Mixed Language blocks. Two color cues were equally presented (e.g., eight pink and eight blue) in alternating order during this block.

**Figure 2:**
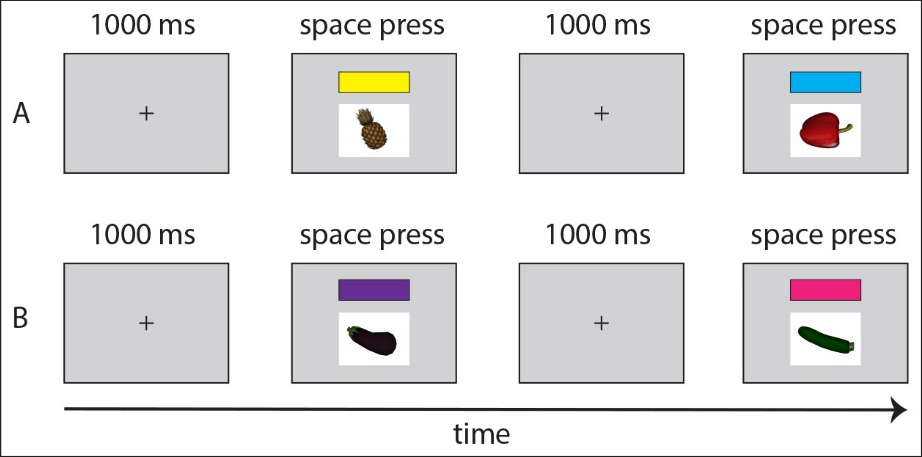
**A:** Two example trials from the Language A Practice Block. For half of the participants, Language A corresponded to English. As such, they named each presented item in English, before pressing the space bar on a keyboard to move to the next trial. **B:** Two example trials from a corresponding Language B Practice Block for the same participant. If Language B indeed corresponded to Dutch for a participant, they were required to name each of the presented items in Dutch. Note that this block also familiarized participants with the link between color cues and the to-be-used languages.

Trials in this block consisted of a fixation cross (1s), followed by the concurrent presentation of a picture (600 × 450 pixels) and a color cue (600 × 250 pixels) above that picture (see Figure 2). Participants were instructed to name the picture in the language of this block and press the spacebar on a keyboard to move to the next trial or ask the experimenter for the correct name of the picture in case they were unsure of its name. Participants received informal feedback from the experimenter for each incorrect response. As such, because the goal of this block was to further familiarize participants with the item names and color cues, no response times or performance were measured.

This block was preceded by eight practice trials to familiarize participants with the task. These trials consisted of the four cognate items, repeated twice for a total of eight trials, in a fixed order. After completing these eight trials, participants were given the opportunity to ask the experimenter any questions before continuing to the target trials.

##### Language A Baseline Block: Full Sentence Picture Naming in Single Language Context

The goal of this block was to collect baseline data to later compare with participants’ performance in the mixed block and allow for calculating potential mixing costs. At the start of the block, participants were presented with the basic sentence structure they should respond with in the baseline and mixed blocks. An example sentence using a practice item was provided in written form in either English (“The apple costs 80 cents”) or Dutch (“De appel kost 80 cent”) depending on the to-be-used language in this block (Dutch-only or English-only, counterbalanced across participants, always the same as in the preceding Practice Block). We expected participants to activate the required sentence structure at the start of a trial, before they filled the open slots with the required, variable information (i.e., a label for the presented object, and its respective price). Even though the sentence structure was repetitive, there was some overlap with language production outside the lab in that a structure is retrieved before words are selected to fill up slots in that structure (Levelt, 1989). Here, we note that the sentences’ matrix structure and the object prices, but not the item names, contain several Dutch-English cognate words, an aspect of the experiments that will be discussed in the General Discussion.

Subsequently, participants responded with full sentences as a function of the picture presented on the screen. For example, during the English Baseline Block, a picture of an *eggplant* with a *yellow* color cue could appear on the screen. Participants responded in English by saying, “The eggplant costs 50 cents”. During the Dutch Baseline Block, the same item paired with for instance a purple color cue led participants to say the Dutch equivalent of “The eggplant costs 50 cents”. Participants’ speech was continuously recorded by a wireless Sennheiser microphone. RTs reflected the interval between the presentation onset of the item and participants’ speech onset. Note that also the link between color cues and response language was fully counterbalanced across participants.

**Figure 3:**
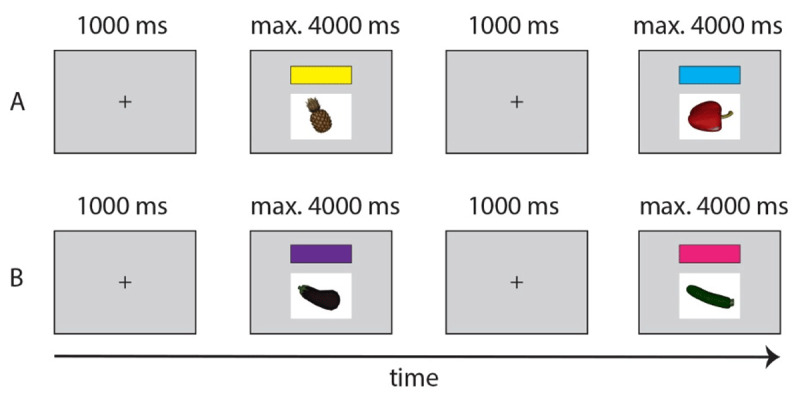
Trial structure in Language A Baseline Block and Language B Baseline Block. Each of these blocks required the use of only one language. A: If yellow and blue color cues referred to Dutch for a participant, they produced sentences in Dutch in response to the presented cue and item. B: For that same participant, purple and pink color cues then required sentence production in English. The relation between to-be-used language and corresponding color cues was counterbalanced across participants.

An individual trial in this block began with a fixation cross (1s), followed by the simultaneous presentation of a picture of one of the items and one of the two color cues matched with that language for a participant directly above the picture (see Figure 3). The picture disappeared 2s after the detection of speech onset or remained on the screen for a maximum of 4s if the voice key was not activated. This inter-trial interval was chosen to have the same timing as in Experiment 2, where it allowed participants’ gaze to go back to baseline and allow for a new virtual agent to appear (see below).

In this block, 16 items were repeated four times for a total of 64 trials. Items were presented in random order and color cues switched after each trial. The baseline block was followed by a short break (for a minimum of 60 seconds) until participants felt comfortable to move to the Language B Practice Block.

##### Language B Practice Block: Single-Word Picture Naming and Color Cue Familiarization

This block was identical to the Language A Practice Block, except for the to-be-used language. Participants first briefly reviewed a booklet with pictures of all 20 items and their names in the respective language (Dutch or English), after which they named each picture once in the to-be-used language. For half of the Dutch-English participants, Language B referred to their L2 English; for the other half the participants, Language B referred to their L1 Dutch.

##### Language B Baseline Block: Full Sentence Picture Naming in Single Language Context

This block was identical to the Language A Baseline Block, except for the to-be-used language. Participants were hence first presented with the to-be-used sentence structure in the respective language, and then produced full sentences as a function of the color cue presented above a picture, the presented item and its price. For half of the participants, Language B referred to their L2 English; for the other half of the participants, Language B referred to their L1 Dutch. For all participants, the to-be-used language was the same as in the preceding block. Participants were given a short break of at least 60 seconds before beginning the final block of the experiment.

##### Mixed Language Block

**Figure 4:**
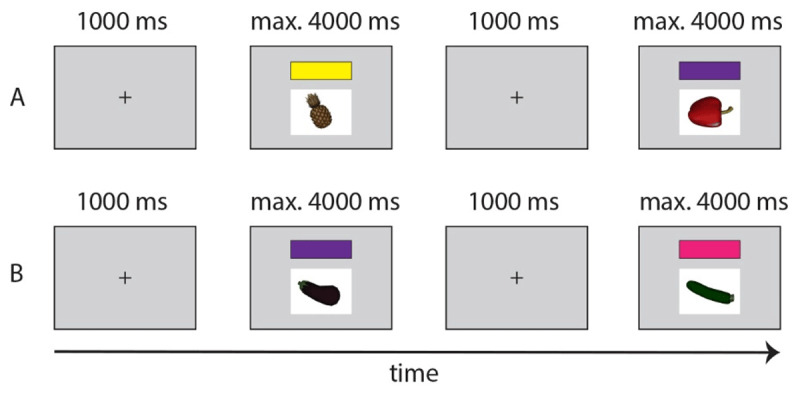
**A:** Language switch sequence in case the yellow and purple color cues corresponded to different languages. **B:** Language repeat sequence in case the purple and pink color cues corresponded to the same language.

The mixed block had the same trial structure as the baseline blocks, but mixed both languages using one of four pseudorandomized lists. Specifically, participants responded in Dutch or English using a full sentence as a function of the presented picture and one of the four language cues (see Figure 4). The match between color cues (two per language) and language (Dutch or English) was counterbalanced across participants to create a total of 24 unique list combinations. In this block, participants were shown each of the 16 images four times in each of the four conditions, leading to a total of 256 trials. Each item was presented an equal number of times with each color cue (i.e., four times). The same image or color cue was never shown twice in a row and the same language was not prompted on more than a maximum of six consecutive trials. As such, both switch trials and repeat trials always came with a switch in color cue. Participants were given a break midway through the block (following trial 128), before continuing the second half of this block. RTs again reflected the interval between the presentation onset of the item and participants’ speech onset.

##### L2 English Proficiency and Inhibitory Control Tests

Following the completion of the experiment, participants first performed the English LexTALE task (Lemhöfer & Broersma, 2012). LexTALE is a short and standardized vocabulary test that here provided a general measure of English vocabulary knowledge as a proxy for participants’ proficiency in their second language English. LexTALE consists of 40 real words and 20 pseudowords. Specifically, participants were shown a string of letters (between 4 and 12 letters long) on a computer screen and had to decide if the string was an English word or not by pressing one of two buttons.

Next, participants completed the *AX*-CPT Baseline task (taken from Gonthier et al., 2016) as it allowed us to calculate a measure (i.e., Proactive Behavioral Index Response Times or *PBI-RTs*) of their general (reactive and proactive) inhibitory control skills (cf. Braver et al., 2009). In this computer task, participants were instructed to press a target button when a cue letter A was followed by a probe letter X. Alternative letter sequences (AY, BX, BY) required pressing a different button. The PBI is calculated via the following formula (AY-BX)/(AY+BX). A positive PBI is typically taken to indicate a participant’s use of proactive control, whereas a negative PBI can be taken as an indication of reactive control (Gonthier et al., 2016). We used the scripts provided by and the exact procedures described by Gonthier and colleagues (2016) to calculate the PBI-RT measure for each participant.

The L2 proficiency (LexTALE score) and inhibitory control (PBI-RT) scores allowed us to check whether there were any baseline differences in L2 proficiency and inhibitory control skills across the different bilingual participant groups taking part in Experiments 1 and 2. Finally, participants filled out a language background questionnaire (LHQ3, taken from Li et al., 2020) to self-report their proficiency in Dutch and English.

#### Participant Exclusion and Data Analyses

Participants were excluded from analysis and replaced on three grounds. First, in line with earlier studies that used a similar design (Peeters, 2020; Peeters & Dijkstra, 2018), any participant for whom more than 25% of the total trials from the Baseline and Mixed blocks needed to be rejected from the RT analyses (e.g., due to technical issues, errors, hesitations, or incorrect responses) were excluded from analysis and replaced. Second, any participant who obtained a score below 48 on the LexTALE English proficiency task (Lemhöfer & Broersma, 2012) was excluded from analysis and replaced.^1^ This score was based on the B1 level from the LexTALE assessment. Previous studies drawing samples from the same bilingual population observed average LexTALE English scores of approximately between 75 and 80 (Peeters, 2020; Peeters & Dijkstra, 2018). Third, participants not reaching at least 80% correct in the Price Memorization Block were excluded from further participation and replaced (see above). In total, data from two participants was discarded (1 due to making too many errors, 1 due to being bilingual from birth). These were replaced by two new participants.

All behavioral data was pre-processed and analyzed in R (Version 3.4.1; R Core Team, 2017). Only the data recorded during the Baseline Blocks and the Mixed Block was analyzed. First, data from all practice trials, the first trial of a block, as well as the first trial after each midway break were excluded. Second, prior to analysis, incorrect responses were removed from the RT dataset. Incorrect responses were defined as trials on which i) the participant responded in the incorrect language, ii) the item was named incorrectly, iii) no response was recorded within the 4s response window, iv) a false start, hesitation, or speech error was observed, or v) an incorrect sentence structure was used. Trials categorized as i), ii), or iv) were considered errors and included as such in the error analyses. As a sanity check, we measured the duration of the produced definite determiner and the duration of the pause between determiner and noun on each trial to be able to make sure no potential differences in RT across conditions related to noun retrieval were obscured by our sentence-onset dependent RT measure.

In terms of data trimming, all RT outliers were removed from the dataset prior to RT analyses. An outlier was defined as an RT that was more than 2.5SD away from that participant’s average RT on correct trials within a block, exceeded the response deadline of 4000 ms, or was found to be below 400 ms as this likely reflected a spillover from the previous trial. An inverse transform (–1000/RT) was used on the RT data to reduce non-normality. With the remaining dataset of 10,595 trials in total, we examined the RTs for switch costs, mixing costs, and reversed language dominance with linear mixed-effects models using the lme4 package (Version 1.1.13, Bates et al., 2014). We further tested for possible switch costs, mixing costs, and reversed language dominance using a logistic mixed effects regression analysis on the error rates.

Two 2 × 2 linear mixed effects models (*lmer*) were run to examine potential switch costs, reversed language dominance, and mixing costs in the RT data. A first model (“RT Switch Cost Model”) included factors Language (L1, L2) and TrialType (Language Switch, Language Repeat) in an RT analysis of the mixed block. A significant main effect of TrialType, and longer RTs for language switch trials compared to language repeat trials, was taken to indicate the presence of switch costs. A significant main effect of Language, and longer RTs for L1 trials compared to L2 trials, was taken to indicate a reversed language dominance. The absence of a significant Language × TrialType interaction, as in previous studies testing samples from this population (Peeters, 2020; Peeters & Dijkstra, 2018), was taken to indicate that switch costs were statistically symmetrical across the two languages.

A second model (“RT Mixing Cost Model”) was used to test for mixing costs and included factors Language (L1, L2) and TrialType (Single Language, Language Repeat) in an RT analysis of the language repeat trials from the mixed block (*n* = 128 trials per participant) and all trials from the baseline blocks (*n* = 128 trials per participant). A significant main effect of TrialType, and longer RTs for language repeat trials compared to trials from the single-language blocks, was taken to indicate the presence of mixing costs. A significant main effect of Language, and longer RTs for L1 trials compared to L2 trials, was again taken to indicate a reversed language dominance. The presence of a significant Language × TrialType interaction, as in previous studies testing samples from this population (Peeters, 2020; Peeters & Dijkstra, 2018), could indicate that mixing costs were statistically larger for the L1 compared to the L2.

Mixed effect models on the error rates exactly mimicked the models used in the RT analyses as shown in Table 2, but took the accuracy (correct response: 0; incorrect response: 1) per trial per participant as input data, rather than their RT, using a logistic approach to data analysis through the *glmer* function.

**Table 2:** Linear mixed effects models that we pre-registered to use in the analyses of the response time data collected in Experiment 1 (Switch Cost Model and Mixing Cost Model).


RT Switch Cost Model Experiment 1: ReactionTime ~ Language*TrialType + (1 + Language*TrialType | Subject) + (1 + Language*TrialType | Item)

RT Mixing Cost Model Experiment 1: ReactionTime ~ Language*TrialType + (1 + Language*TrialType | Subject) + (1 + Language*TrialType | Item)


*Note*: RT Switch Cost Model: Language (Dutch = –0.5; English = 0.5) and TrialType (Language Repeat = –0.5 and Language Switch = 0.5) were sum-coded. RT Mixing Cost Model: Language (Dutch = –0.5; English = 0.5) and TrialType (Language Repeat = –0.5 and Single-Language = 0.5) were sum-coded.

Additionally, in case any of the statistical models did not converge, we simplified the random effects structures by using the *buildmer* package (Voeten, 2020). Specifically, this package allowed for the automatic and systematic simplification of random slopes by finding the largest (possible) regression model that converged.

Raw data, analysis scripts, laboratory log, and a full pre-registration of the present study can be found on the OSF via: https://osf.io/5qp8x/?view_only=9ebc812967fc4d5abf0ce66147eee315.

## Results

### RT results

**Figure 5:**
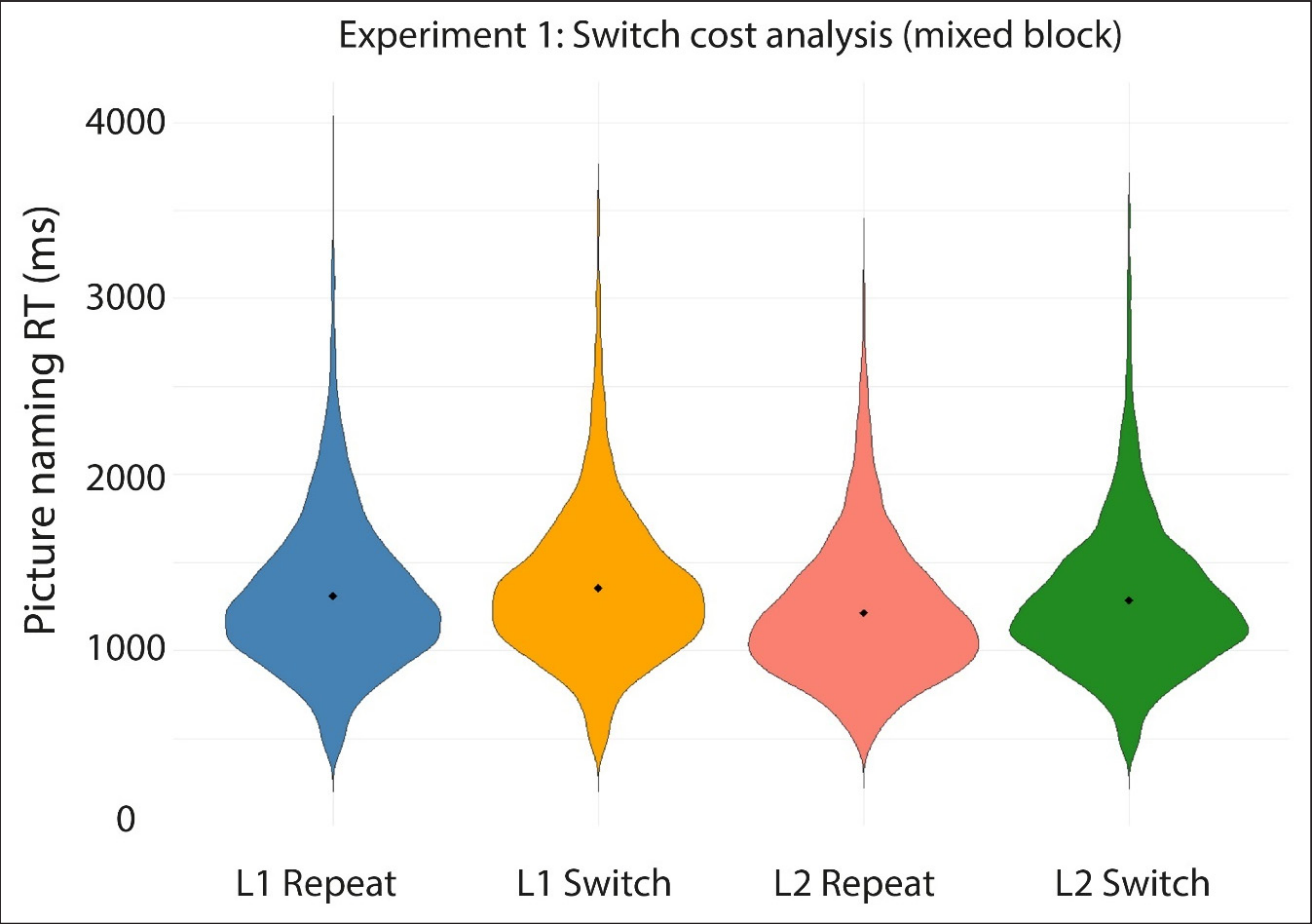
Violin plots depicting the RT data on correct trials in each of the four conditions in the mixed block in Experiment 1. Filled diamonds indicate the average RT for each condition.

**Figure 6:**
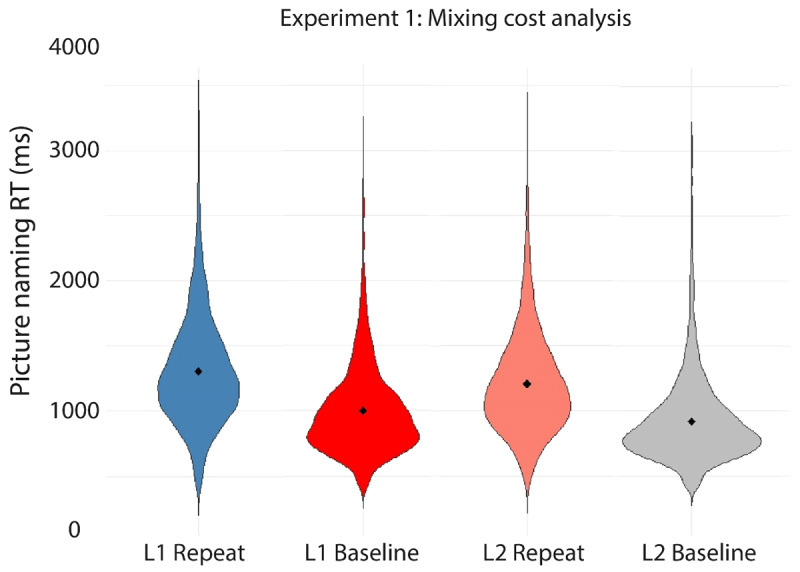
Violin plots depicting the RT data on correct trials in each of the four conditions in the mixing cost analysis in Experiment 1. Filled diamonds indicate the average RT for each condition.

Table 3 and Figures 5 and 6 present the mean RTs per condition in the experiment. Table 4 presents the outcome of the linear mixed effects analyses conducted on these data.

**Table 3:** Average reaction times (in ms) and error rate (in proportions) per condition in Experiment 1. Values within parentheses are standard deviations.


CONDITION	RT	ERROR RATE

L1 (Dutch) Baseline	998 (351)	.036 (.186)

L2 (English) Baseline	913 (314)	.023 (.150)

L1 (Dutch) Language Switch	1354 (431)	.105 (.307)

L1 (Dutch) Language Repeat	1310 (433)	.072 (.259)

L2 (English) Language Switch	1284 (419)	.065 (.246)

L2 (English) Language Repeat	1214 (401)	.033 (.180)


**Table 4:** Outcome of the linear mixed effects models performed on the RT data from Experiment 1. Model structure reflects the model fit by maximum likelihood as indicated by the *buildmer* package. Significant *p* values are indicated in boldface.


**1. Mixed Block comparison**				

RT Switch Cost Model: ReactionTime ~ Language*TrialType + (1+Language*TrialType | Subject) + (1+Language*TrialType | Item)				

	Estimate	SE	*t* value	*p* value

Language	–91.31	15.74	–5.80	**1.62e** ^-06^

TrialType	57.37	8.06	7.12	**8.62e** ^-07^

Language × TrialType	23.52	17.50	1.34	0.19

**2. Mixing Cost analysis**				

RT Mixing Cost Model: ReactionTime ~ Language*TrialType + (1 + Language*TrialType | Subject) + (1 + Language*TrialType | Item)				

	Estimate	SE	*t* value	*p* value

Language	–90.31	16.98	–5.32	**3.76e** ^-06^

TrialType	–303.95	24.97	–12.17	**4.00e** ^-16^

Language × TrialType	8.51	19.96	0.43	0.67


*Note*: RT Switch Cost Model: Language (Dutch = –0.5; English = 0.5) and TrialType (Language Repeat = –0.5 and Language Switch = 0.5) were sum-coded. RT Mixing Cost Model: Language (Dutch = –0.5; English = 0.5) and TrialType (Language Repeat = –0.5 and Single-Language = 0.5) were sum-coded.

Using the first model specified in Table 4, we first tested for effects of Language (L1 Dutch, L2 English), TrialType (Language Switch, Language Repeat), and their interaction on the RTs collected in the mixed block. We observed a significant main effect of TrialType, indicating that switch trials (*M* = 1318 ms) yielded significantly longer RTs than repeat trials (*M* = 1260 ms). In addition, a significant main effect of Language indicated that RTs on L1 Dutch trials (*M* = 1332 ms) were significantly longer than RTs on L2 English trials (*M* = 1248 ms). No interaction between TrialType and Language was observed. In sum, we observed the expected pattern of symmetrical switch costs and reversed language dominance (see Figure 5).

Additionally, using the second model specified in Table 4, the trials from the single language baseline blocks (i.e., L1-only or L2-only) were compared to the language repeat trials from the mixed block to test for the potential presence of mixing costs in the RTs. A significant main effect of TrialType indicated that RTs were significantly longer for the language repeat trials in the mixed block (*M* = 1249 ms) compared to the trials in the single-language blocks (*M* = 956 ms). In addition, a significant main effect of Language confirmed the reversed language dominance observed above, as participants were again shown to be slower in their L1 Dutch (*M* = 1141 ms) compared to their L2 English (*M* = 1061 ms). Finally, no significant interaction between TrialType and Language was observed. In sum, we observed symmetrical mixing costs and a reversed language dominance in the mixing cost RT analysis (see Figure 6).

### Accuracy results

Trials on which the incorrect language was used, the depicted item was named incorrectly, or a false start, hesitation, or speech error was observed were considered errors. The average error rate for each experimental condition can be found in Table 3.

In the logistic mixed effects analysis performed on the error rate data from the mixed block (first model Table 5), we firstly observed a main effect of Language, indicating that participants made significantly more errors in Dutch (*M* = .089) compared to English (*M* = .049). We also observed a main effect of TrialType, indicating that participants made more errors during switch trials (*M* = .085) compared to repeat trials (*M* = .053). Finally, we did not observe a significant interaction effect between Language and TrialType. In sum, also in the accuracy results, we observed symmetrical switch costs and a reversed language dominance in the mixed block.

**Table 5:** Outcome of the logistic mixed effects models performed on the error rate data from Experiment 1. Model structure reflects the model fit by maximum likelihood as indicated by the *buildmer* package. Significant *p* values are indicated in boldface.


**1. Mixed Block comparison**				

Accuracy Switch Cost Model: ErrorRate ~ 1 + Language*TrialType + (1 + TrialType | Subject) + (1 + Language | Item)				

	Estimate	SE	*z* value	*p* value

Language	–.70	.12	–5.92	**3.25e** ^–09^

TrialType	.59	.10	5.69	**1.26e** ^–08^

Language × TrialType	.26	.16	1.61	.11

**2. Mixing Cost analysis**				

Accuracy Mixing Cost Model: ErrorRate ~ 1 + Language*TrialType + (1 + TrialType | Subject) + (1 + Language | Item)				

	Estimate	SE	*z* value	*p* value

Language	–.21	.15	–1.44	.15

TrialType	–.54	.15	–3.48	**.0005**

Language × TrialType	1.31	.20	6.41	**1.44e** ^–^^10^


*Note*: Accuracy Switch Cost Model: Language (Dutch = –0.5; English = 0.5) and TrialType (Language Repeat = –0.5 and Language Switch = 0.5) were sum-coded. Accuracy Mixing Cost Model: Language (Dutch = –0.5; English = 0.5) and TrialType (Language Repeat = –0.5 and Single-Language = 0.5) were sum-coded.

Additionally, using the second model specified in Table 5, the trials from the single language baseline blocks (i.e., L1-only or L2-only) were compared to the language repeat trials from the mixed block to test for the potential presence of mixing costs in the error rates. We observed a significant main effect of TrialType, indicating that participants made more errors on language repeat trials in the mixed block (*M* = .053) compared to single-language trials in the baseline blocks (*M* = .029). In addition, although participants were numerically found to make more errors in Dutch (*M* = .046) compared to English (*M* = .034), no significant main effect of Language was observed. Finally, in this analysis, we observed a significant interaction between TrialType and Language. Separate follow-up logistic mixed effects analyses per language indicated a main effect of TrialType for the Dutch trials (Est. = –1.19, SE = .22, *z* = –5.51, *p* = 3.62e^–08^), but no main effect of TrialType for the English trials (Est. = .16, SE = .30, *z* = .53, *p* = .60). In sum, we observed significant asymmetrical mixing costs in the mixing cost analysis on the error rates.

## Interim Discussion

In Experiment 1, a group of relatively proficient Dutch-English bilinguals produced sentences in either their L1 Dutch or their L2 English on the basis of a picture presented on a computer screen and as a function of an arbitrary color cue that indicated which language to use. Five experiments testing different participant samples from this population previously yielded robust symmetrical switch costs, reversed language dominance, and asymmetrical mixing costs (Peeters & Dijkstra, 2018; Peeters, 2020). In these earlier studies, participants named pictures in single words in a classic cued language-switching paradigm (cf. Costa & Santesteban, 2004; Meuter & Allport, 1999) that was administered on a computer screen or in virtual reality. The present experiment shows that, across the board, the results we observed earlier for this population generalize to situations where these bilinguals produce full sentences rather than single words.

Indeed, in Experiment 1, a cognitive cost was reflected in both longer RTs and higher error rates when participants switched between languages compared to when they were cued to stay in the same language on consecutive trials. As in the earlier studies on this population, this cost was statistically similar across the two languages. The switch cost as a marker of reactive, trial-by-trial inhibition was accompanied by two markers of proactive, sustained inhibition of the L1: a reversed language dominance in both RTs and error rates throughout the experiment, and larger mixing costs for the L1 compared to the L2 in the error rates. These findings hence suggest that, also when it comes to the production of sentences, language production in this population of Dutch-English bilinguals in an overall dual-language context is supported by both reactive and proactive inhibitory control mechanisms (Green & Abutalebi, 2013).

Having replicated the predicted result pattern in a sentence context, Experiment 1 can serve as the perfect baseline for Experiment 2 in which a different sample of participants from our Dutch-English bilingual population will be immersed in a visually rich 3D marketplace scenario in virtual reality. In addition to the increased visual richness of the experimental environment, also the communicative relevance of the participants’ utterances will be enhanced, as bilingual participants will produce sentences in Dutch or English as a function of the language background of the monolingual visitor of their market stand. Nevertheless, to allow for a valid comparison, the sentences participants will produce will be the same as these produced in Experiment 1. Will the increase in naturalness of the setup change the pattern of results consistently observed in six earlier experiments (i.e., the present study’s Experiment 1; Peeters, 2020; Peeters & Dijkstra, 2018)?

## Experiment 2

### Method

#### Participants

Forty-eight L1 speakers of Dutch (*M* age = 23.6; age range: 18–29 years old; 31 female, 17 male) participated in this experiment, matched in age and language background with the participants in Experiment 1. None of these participants took part in Experiment 1. They were financially compensated for their time (10 euros per hour). Data from 5 additional participants was recorded but discarded, due to technical failure (3 participants) or making too many errors (2 participants).

#### Stimuli

The 20 pictures (Exp. 1) were replaced by 20 3-D objects corresponding to the items presented in the pictures. The 3-D objects were designed by a graphic designer using Autodesk Maya software and partially taken from the standardized database of 3-D objects provided by Peeters (2018). The four color cues (Exp. 1) were replaced by four virtual agents (two male, two female), of which two represented a Dutch cue and the other two represented an English cue. The virtual agents had distinctive facial features (e.g., glasses, hair color, hair length, gender) and clothing (see Appendix B). Finally, rather than being placed in a soundproof booth (Exp. 1), participants were immersed in a virtual marketplace, where they spoke to each of the virtual agents. Further detail about the display and virtual agents can be found in the *Display* and *Procedure* sections below.

#### Display

Experiment 2 took place in a CAVE environment (Cruz-Neira et al., 1993). The environment used six projectors that projected a virtual marketplace environment onto three large screens (255 cm × 330 cm, VISCON GmbH, Neukirchen-Vluyn, Germany), using two projectors per screen. Participants wore active 3-D shutter glasses, in order to become immersed in the virtual environment. This CAVE setup has been described in detail by Eichert et al. (2018). The experiment was programmed and run using Vizard software (Vizard, Floating Client 5.4, WorldViz LLC, Santa Barbara, CA). The speech from each virtual agent was presented from two speakers that were arranged at the bottom of the screen that faced the participants.

#### Procedure

Experiment 2 consisted of seven main stages that were kept as similar as possible to Experiment 1: i) a price memorization block in which participants learned the price of each object presented in the experiment, ii) a first practice block aimed at practicing the item names in Language A (e.g., Dutch), iii) a baseline block in which participants used full sentences in Language A (e.g., Dutch), iv) a second practice block in which participants practiced using the item names in Language B (e.g., English), v) a second baseline block in which participants used full sentences in Language B (e.g., English), vi) a mixed block in which participants produced full sentences while both languages were intermixed across trials, vii) a separate set of control tests that included the LexTALE English proficiency test (Lemhöfer & Broersma, 2012), the *AX*-CPT Baseline task (taken from Gonthier et al., 2016), and the language proficiency questionnaire (LHQ3, taken from Li et al., 2020).

##### Price Memorization Block

The goal of this block was to familiarize participants with the link between the objects and their prices. Participants were immersed in an adapted stock environment from WorldViz (“room.wrl”) in the CAVE environment. This 3-D environment represented a virtual room that contained a virtual computer monitor (see Figure 8). The same procedure was used as in the Price Memorization Block in Experiment 1, but using the virtual environment to present the objects (on a virtual computer screen that mimicked the real computer screen in Experiment 1) and prices (again presented in text to the bottom left and bottom right of the object, on the virtual computer screen). Participants responded by using an X-Box controller and pressing either the top left bumper or top right bumper of the controller. All other aspects of the block matched the corresponding block in Experiment 1.

##### Language A Practice Block: Single-Word Picture Naming and Virtual Agent Familiarization

At the start of this block, two of the four virtual agents were introduced to the participant in the CAVE environment to familiarize them with which language to respond with to which virtual agent. The virtual agents acted as relatively natural language cues as opposed to the arbitrary color cues in Experiment 1. The introduction of the virtual agents was as follows. If this block was an L1 Dutch block for a specific participant, two virtual agents (one male, one female) introduced themselves in Dutch and stated that they were only able to speak Dutch. For example, they said the Dutch equivalent of “Hello. My name is Marloes and I am only able to speak Dutch. Whenever I appear in front of you, please only talk to me in Dutch.” They looked at the participant and their mouth moved as a function of the amplitude of their speech (“lip-sync”). The other Dutch virtual agent repeated this introduction in Dutch, but with a different name (e.g., Jan) and voice. If this block was an English block for a specific participant, the two virtual agents (one male, one female) introduced themselves in a similar way in English and with two typically English names (i.e., Alice and Jake). These sentences were pre-recorded from L1 speakers of Dutch and English respectively, who matched the virtual agents in age and ethnicity. We considered the virtual agents’ spoken introduction relatively similar to an everyday situation in which one meets a new person and first negotiates in which language to speak.

Following the introduction of the two virtual agents, participants familiarized themselves with the items and the relation between virtual agents and language background to practice for subsequent blocks. The set-up was identical to the setup in Experiment 1, except that the virtual agents (as opposed to color cues) served as the language cues. This block took place in the same virtual environment as used in the previous block. In the environment, the virtual room did however now also contain a virtual agent placed behind the virtual computer monitor, with only part of their chest and all of their neck and head showing above the monitor (see Figure 9). With this set-up, we were able to use a relatively similar position of the language cues compared to Experiment 1. All other aspects of the Language A Practice Block were identical to Experiment 1.

##### Language A Baseline Block: Full Sentence Object Naming in Single Language Context

**Figure 7:**
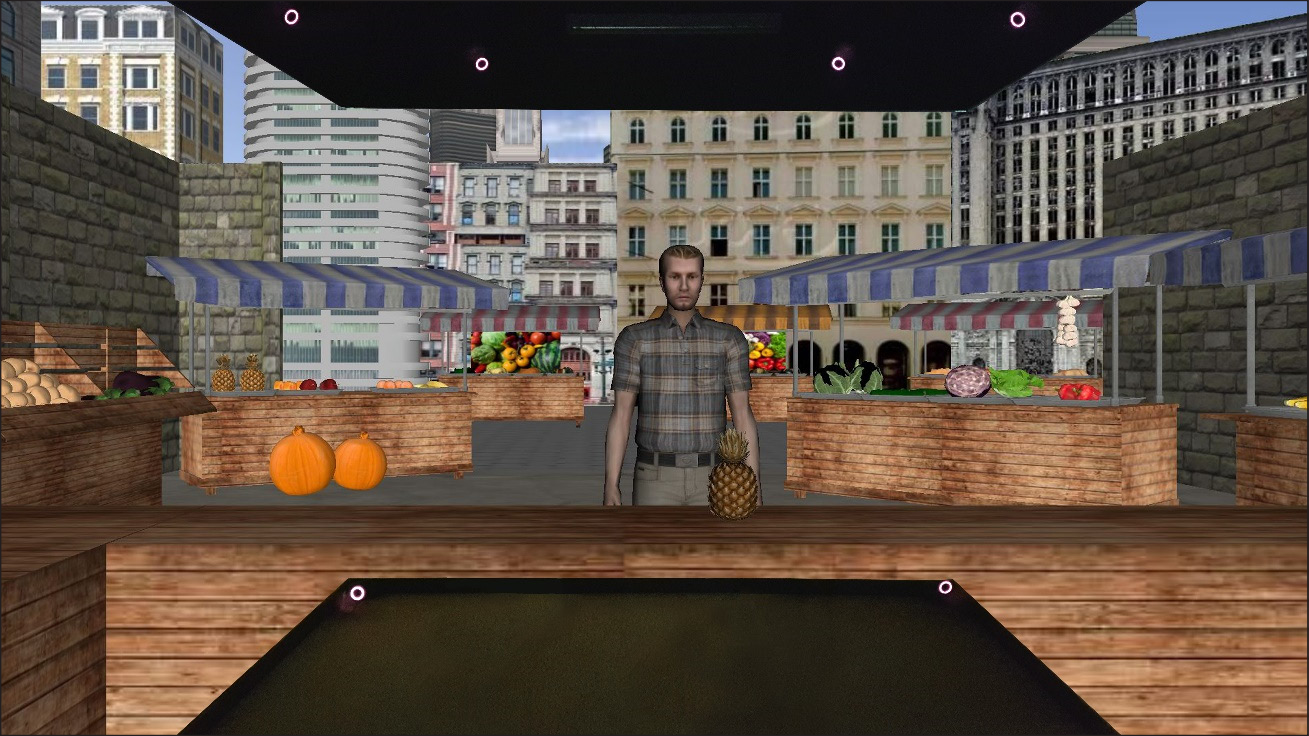
Illustration of the CAVE setup with its three connected screens. Participants were immersed in a virtual marketplace and acted as a storeowner of a fruit and vegetables stand. Virtual agents (one at a time, as depicted here) visited the stand. A subset of the infrared-cameras that allowed for motion tracking are present as red circles in the picture.

The goal of this block was to collect baseline data to later compare with the mixed block to calculate potential mixing costs. All aspects were similar to the corresponding block in Experiment 1, except for the visual environment presented to the participants. In this block, participants were immersed in a rich marketplace setting in the CAVE environment (see Figure 7). This was done to partially replicate a typical everyday situation, where a bilingual would be likely to use a certain language as a function of the language background of their interlocutor in a broader, situated environment. The participant acted as a storeowner in a marketplace and spoke to the virtual agents who appeared in front of them. Specifically, an item appeared at the same time as a virtual agent on the other side of the table of the market stand. We considered this relatively similar to a natural situation in which someone might approach an owner of a market stand to ask about the cost of a fruit or vegetable. The item to which the participant referred was placed in central position on the virtual table, similar to the position of the image in Experiment 1 on the computer screen (cf. Figure 7).

**Figure 8:**
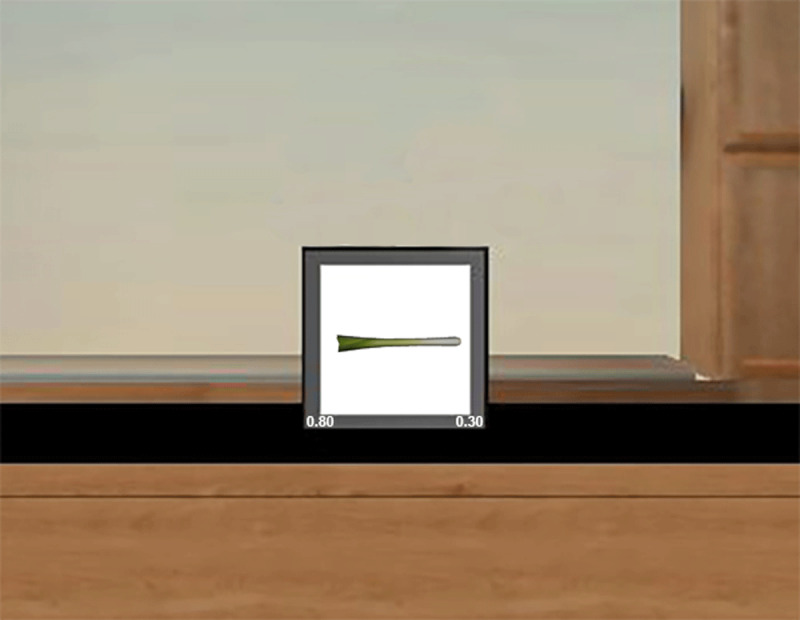
In the Price Memorization Block in Experiment 2, participants practiced the link between the fruit and vegetable items and their prices. They indicated via button press what price they thought an object had, selecting either the left or right option as presented at the bottom of the virtual computer screen as depicted here, and received on-screen feedback after their response, as in Experiment 1.

**Figure 9:**
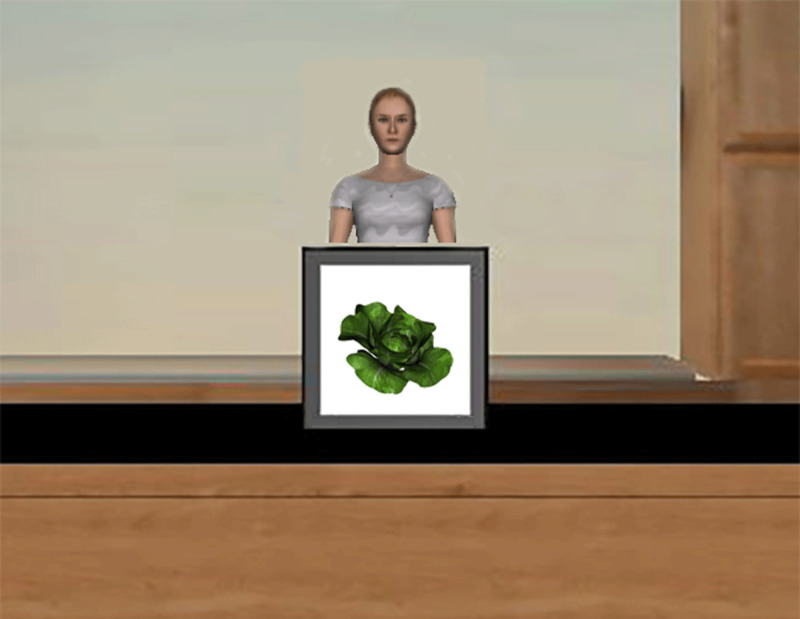
In the Language Practice Blocks in Experiment 2, participants named each of the pictures once in Dutch (in the Dutch block) and once in English (in the English block). Per practice block, two virtual agents were presented (one at a time) to further familiarize participants with the link between language cue (i.e., a virtual agent) and the language they should respond with (Dutch for two virtual agents, English for two other virtual agents).

In sum, participants told the visitors of their stand what each item cost in a full sentence (e.g., “The eggplant costs 50 cents”), answering in their L1 Dutch or L2 English as a function of the language identity of the virtual agent that faced them. RTs reflected the interval between the presentation onset of the item and participants’ speech onset.

##### Language B Practice Block: Single-Word Picture Naming and Virtual Agent Familiarization

This block was identical to the Language A Practice Block used in Experiment 2, except for the to-be-used language. Participants hence first briefly reviewed a booklet with pictures of all 20 items and their names in the respective language (Dutch or English), after which they named each picture once in the to-be-used language.

##### Language B Baseline Block: Full Sentence Picture Naming in Single Language Context

This block was identical to the Language A Baseline Block used in Experiment 2, except for the to-be-used language. Participants hence first learned the to-be-used sentence structure in the respective language, and then produced full sentences as a function of the language background of the virtual agent, the presented 3-D item, and its price.

##### Mixed Language Block

All aspects of the virtual environment remained the same as in the previous block. Procedurally, all aspects replicated those described in the Mixed Block of Experiment 1. As such, participants told the visitors of their stand what each item cost in a full sentence, mixing between their L1 Dutch and L2 English as a function of the language identity of the virtual agent that appeared in front of them. RTs again reflected the interval between the presentation onset of the item and participants’ speech onset. Note that target stimulus (the 3-D object) and language cue (the virtual agent) became visible to the participant at the same time, as in Experiment 1 and as in all Baseline Blocks, such that there was no room for differential, reactive language pre-activation patterns between experiments and blocks.

##### L2 English Proficiency and Inhibitory Control Tests

As in Experiment 1, participants took the English LexTALE test (Lemhöfer & Broersma, 2012), the *AX*-CPT Baseline task (Gonthier et al., 2016), and filled out the language background questionnaire (Li et al., 2020) to self-report their proficiency in Dutch and English.

#### Participant Exclusion and Data Analyses

The procedure of participant exclusion and data analysis was identical to the procedure used in Experiment 1, except that, in the VR set-up, participants’ utterances were recorded in one long audio file that included markers for each trial onset, such that RTs could be calculated off-line using Parselmouth (Jadoul et al., 2018), a *python* interface to Praat (Boersma & Weenink, 2021). In addition to the analysis of the data from Experiment 2, we subsequently carried out an overall analysis combining the data from the two experiments adding the factor Experiment with two levels (Exp. 1 and Exp. 2) to the statistical models. Any significant interaction of an original main or interaction effect with the factor Experiment was taken to indicate a significant difference in behavior (as reflected by switch costs, reversed language dominance, or mixing costs) between the two setups (traditional computer setup as in Exp. 1 vs. an immersive, communicatively more relevant setup as in Exp. 2).

As no significant difference in average baseline inhibitory control skills was observed between the groups taking part in Experiment 1 vs. Experiment 2, as measured through the *AX*-CPT Baseline task measure, participants’ average PBI-RT was not included as a covariate in the two overall statistical models to account for any baseline variance between the two participant groups. The same held for participants’ LexTALE scores.

The pre-registered mixed effect models on the error rates exactly mimicked the models used in the RT analyses as shown in Table 7, but took the accuracy (correct response: 0; incorrect response: 1) per trial per participant as input data, rather than their reaction time, using a logistic approach to data analysis through the *glmer* function.

**Table 6:** Participant characteristics for Experiment 2: average score on the L2 English LexTALE test, average score on the *AX*-CPT test, self-reported age of acquisition (‘AoA’, in years of age) and proficiency (‘SRP’; based on a 1–7 Likert scale) with regards to listening, speaking, reading, and writing in both L1 Dutch and L2 English, and self-reported average hours of use per day of L2 English. For comparison with Table 1.


MEASURE	AVERAGE	SD

LexTALE	80.3	13.50

*AX*-CPT	–0.03	0.05

L1 Listening AoA	0.2	0.57

L1 Speaking AoA	0.5	0.85

L1 Reading AoA	1.2	2.01

L1 Writing AoA	1.4	2.29

L1 Listening SRP	7.0	0.00

L1 Speaking SRP	7.0	0.00

L1 Reading SRP	7.0	0.00

L1 Writing SRP	7.0	0.00

L2 Listening AoA	9.5	4.39

L2 Speaking AoA	10.8	3.63

L2 Reading AoA	10.5	3.31

L2 Writing AoA	11.3	3.16

L2 Listening SRP	6.1	0.87

L2 Speaking SRP	5.0	1.10

L2 Reading SRP	5.8	1.10

L2 Writing SRP	5.1	1.29

L2 Hours of use per day	5.2	3.57


**Table 7:** Linear mixed effects models that were pre-registered for the analyses of the response time data collected in Experiment 2 (Switch Cost Model and Mixing Cost Model) and the dataset combining the response time data from both experiments (Switch Cost Model Overall and Mixing Cost Model Overall).


RT Switch Cost Model Experiment 2: ReactionTime ~ Language*TrialType + (1 + Language*TrialType | Subject) + (1 + Language*TrialType | Item)

RT Mixing Cost Model Experiment 2: ReactionTime ~ Language*TrialType + (1 + Language*TrialType | Subject) + (1 + Language*TrialType | Item)

RT Switch Cost Model Overall: ReactionTime ~ Language*TrialType*Experiment + (1 + Language*TrialType | Subject) + (1 + Language*TrialType | Item)

RT Mixing Cost Model Overall: ReactionTime ~ Language*TrialType*Experiment + (1 + Language*TrialType | Subject) + (1 + Language*TrialType | Item)


*Note*: RT Switch Cost Model: Language (Dutch = –0.5; English = 0.5) and TrialType (Language Repeat = –0.5 and Language Switch = 0.5) were sum-coded. RT Mixing Cost Model: Language (Dutch = –0.5; English = 0.5) and TrialType (Language Repeat = –0.5 and Single-Language = 0.5) were sum-coded. Overall models: Experiment (Experiment 1 = –0.5; Experiment 2 = 0.5) added into the models on the combined data from both experiments.

Additionally, in case any of the statistical models did not converge, we simplified the random effects structures by using the *buildmer* package (Voeten, 2020). Specifically, this package allowed for the automatic and systematic simplification of random slopes by finding the largest (possible) regression model that converged.

## Results

### RT results

**Figure 10:**
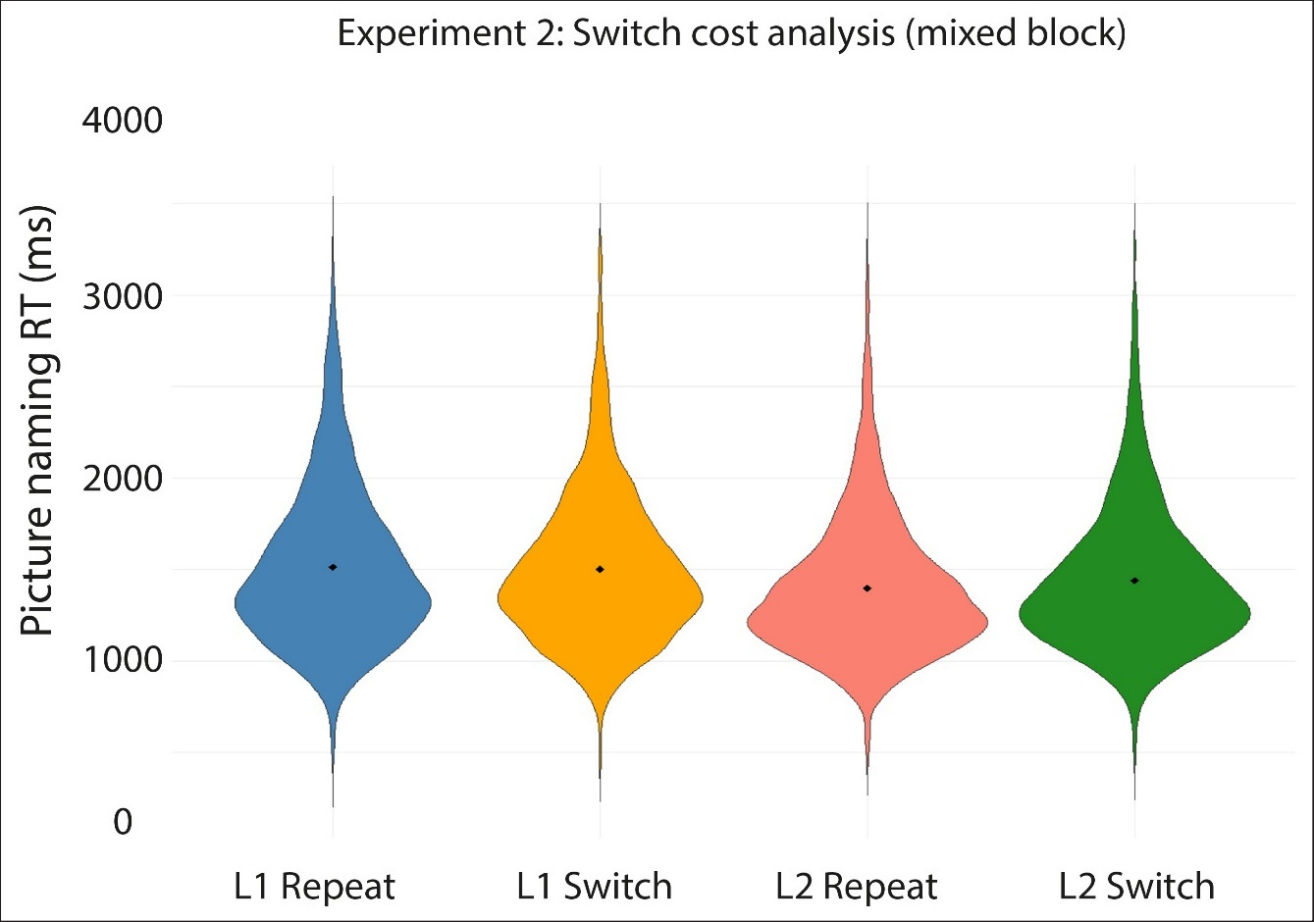
Violin plots depicting the RT data on correct trials in each of the four conditions in the mixed block in Experiment 2. Filled diamonds indicate the average RT for each condition.

**Figure 11:**
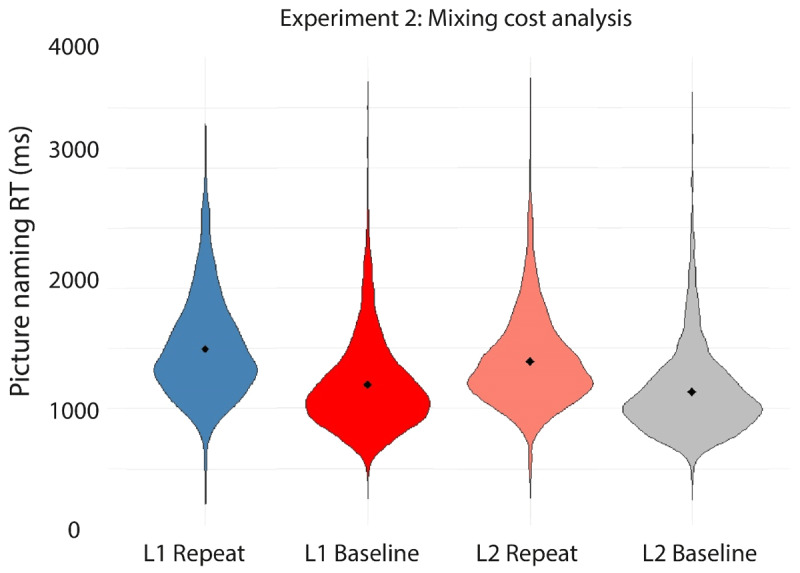
Violin plots depicting the RT data on correct trials in each of the four conditions in the mixing cost analysis in Experiment 2. Filled diamonds indicate the average RT for each condition.

Table 8 and Figures 10 and 11 present the mean RTs per condition in the experiment. Table 9 reports the findings of the linear mixed effects analyses conducted on the RT data.

**Table 8:** Average reaction times (in ms) and error rate (in proportions) per condition in Experiment 2. Values within parentheses are standard deviations.


CONDITION	RT	ERROR RATE

L1 (Dutch) Baseline	1196 (392)	.026 (.160)

L2 (English) Baseline	1137 (367)	.045 (.208)

L1 (Dutch) Language Switch	1502 (409)	.075 (.264)

L1 (Dutch) Language Repeat	1512 (431)	.050 (.219)

L2 (English) Language Switch	1439 (393)	.061 (.239)

L2 (English) Language Repeat	1397 (382)	.042 (.201)


**Table 9:** Outcome of the linear mixed effects models performed on the RT data from Experiment 2. Model structure reflects the model fit by maximum likelihood as indicated by the *buildmer* package. Significant *p* values are indicated in boldface.


**1. Mixed Block comparison**				

RT Switch Cost Model: ReactionTime ~ 1 + Language*TrialType + (1 + Language + TrialType | Subject) + (1 + Language + TrialType | Item)				

	Estimate	SE	*t* value	*p* value

Language	–91.04	16.41	–5.55	**2.07** ^-06^

TrialType	17.88	9.41	1.90	**0.07**

Language × TrialType	48.01	12.16	3.95	**7.91** ^-05^

**2. Mixing Cost analysis**				

RT Mixing Cost Model: ReactionTime ~ Language*TrialType + (1 + Language*TrialType | Subject) + (1 + Language*TrialType | Item)				

	Estimate	SE	*t* value	*p* value

Language	–82.37	17.89	–4.61	**3.46e** ^-05^

TrialType	–275.36	18.90	–14.57	**<2e** ^-16^

Language × TrialType	55.52	20.11	2.76	**0.01**


*Note*: RT Switch Cost Model: Language (Dutch = –0.5; English = 0.5) and TrialType (Language Repeat = –0.5 and Language Switch = 0.5) were sum-coded. RT Mixing Cost Model: Language (Dutch = –0.5; English = 0.5) and TrialType (Language Repeat = –0.5 and Single-Language = 0.5) were sum-coded.

Using the first model specified in Table 9, we first tested for effects of Language (L1 Dutch, L2 English), TrialType (Language Switch, Language Repeat), and their interaction on the RT data from the mixed block. A significant main effect of Language indicated that RTs on L1 Dutch trials (*M* = 1507 ms) were significantly longer than RTs on L2 English trials (*M* = 1418 ms). No significant main effect of TrialType was observed, indicating that RTs on switch trials (*M* = 1470 ms) were not statistically different from RTs on repeat trials (*M* = 1454 ms). We did observe a significant interaction between TrialType and Language. Separate follow-up linear mixed effects analyses per language showed no main effect of TrialType for the Dutch trials (Est. = –6.98, SE = 13.79, *t* = –.51, *p* = .620), while they did show a main effect of TrialType for the English trials (Est. = 42.05, SE = 11.95, *t* = 3.52, *p* = .002). In sum, we observed an overall reversed language dominance paired with switch costs for the L2 English only.

Additionally, using the second model specified in Table 9, the trials from the single language baseline blocks were compared to the language repeat trials from the mixed block to test for the potential presence of mixing costs in the RT data. A significant main effect of TrialType indicated that RTs were significantly longer for the language repeat trials in the mixed block (*M* = 1443 ms) compared to the trials in the single-language blocks (*M* = 1167 ms). In addition, a significant main effect of Language confirmed the reversed language dominance observed above, as participants were again shown to be slower in their L1 Dutch (*M* = 1342 ms) than in their L2 English (*M* = 1263 ms). Finally, a significant interaction between TrialType and Language was observed. Separate follow-up linear mixed effects analyses per TrialType indicated a relatively large main effect of Language for the repeat trials from the mixed block (Est. = –110.23, SE = 19.67, *t* = –5.60, *p* = 2.61e^–06^), but a smaller main effect of Language for the single-language trials (Est. = –54.57, SE = 20.48, *t* = –2.67, *p* = .01). Separate follow-up linear mixed effects analyses per language testing for a main effect of TrialType separately in L1 and L2 did not converge. In sum, we observed significant mixing costs and a reversed language dominance that was larger in the mixed block than in the baseline blocks.

### Accuracy results

Trials on which the incorrect language was used, the object was named incorrectly, or a false start, hesitation, or speech error was observed were considered errors. The average error rate for each experimental condition in Experiment 2 can be found in Table 8.

In the logistic mixed effects analysis performed on the error rate data from the mixed block (first model Table 10), we firstly observed no main effect of Language. We did observe a significant main effect of TrialType, indicating that participants made more errors on switch trials (*M* = .068) than on repeat trials (*M* = .048). Finally, we did not observe a significant interaction effect between Language and TrialType.

**Table 10:** Outcome of the logistic mixed effects models performed on the error rate data from Experiment 2. Model structure reflects the model fit by maximum likelihood as indicated by the *buildmer* package. Significant *p* values are indicated in boldface.


**1. Mixed Block comparison**				

Accuracy Switch Cost Model: ErrorRate ~ 1 + Language*TrialType + (1 + Language | Subject) + (1 + Language | Item)				

	Estimate	SE	*z* value	*p* value

Language	–.32	.21	–1.51	.13

TrialType	.41	.08	4.96	**7.19e** ^-07^

Language × TrialType	–.05	.16	–.29	.77

**2. Mixing Cost analysis**				

Accuracy Mixing Cost Model: ErrorRate ~ 1 + Language*TrialType + (1 + Language | Subject) + (1 + Language | Item)				

	Estimate	SE	*z* value	*p* value

Language	–.16	.27	–.59	.55

TrialType	–.34	.10	–3.46	**.001**

Language × TrialType	.84	.19	4.33	**1.48e** ^-05^


*Note*: Accuracy Switch Cost Model: Language (Dutch = –0.5; English = 0.5) and TrialType (Language Repeat = –0.5 and Language Switch = 0.5) were sum-coded. Accuracy Mixing Cost Model: Language (Dutch = –0.5; English = 0.5) and TrialType (Language Repeat = –0.5 and Single-Language = 0.5) were sum-coded.

Additionally, using the second model specified in Table 10, the trials from the single language blocks (i.e., L1-only or L2-only) were compared to the language repeat trials from the mixed block to test for the potential presence of mixing costs in the error rates. No significant main effect of Language was observed. We observed a significant main effect of TrialType, indicating that participants made more errors on repeat trials during the mixed block (*M* = .046) compared to on trials in the single language blocks (*M* = .036). We also observed a significant interaction between TrialType and Language. Separate follow-up logistic mixed effects analyses per language indicated a main effect of TrialType for the Dutch trials (Est. = –1.62, SE = .0007, *z* = –2359, *p* = 2e^–16^), but no main effect of TrialType for the English trials (Est. = .08, SE = .27, *z* = .29, *p* = .78). In sum, we observed significant asymmetrical mixing costs in the mixing cost analysis on the error rates from Experiment 2.

## Combined analysis Experiments 1 and 2

**Figure 12:**
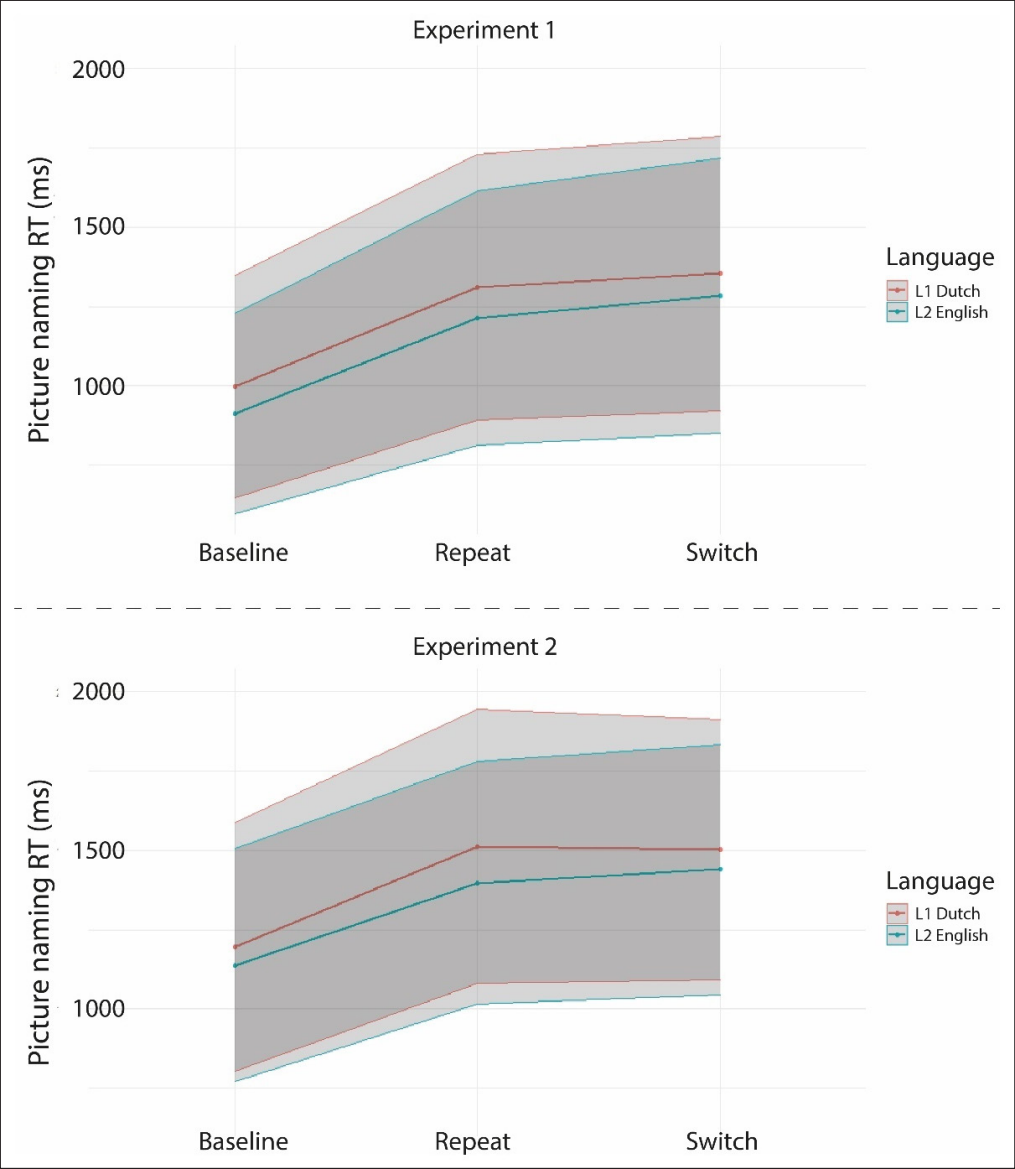
Line graphs depicting the average RT data per condition for Experiment 1 (top panel) and Experiment 2 (bottom panel). Shaded ribbons indicate one standard deviation above and below the mean.

In both experiments, we observed an overall reversed language dominance and robust mixing costs that were mostly larger for the L1 than for the L2. Switch cost patterns, however, clearly differed across the two experiments: while symmetrical switch costs across languages were observed in Experiment 1, in Experiment 2 we found an asymmetry in switch costs in the RTs in that they were present for the L2 English but absent for the L1 Dutch (cf. Figure 12). This difference across experiments was statistically supported by a significant interaction between Language and TrialType in the analysis of the mixed block in Experiment 2, and its absence in Experiment 1. To test for the statistical robustness of this observed difference across experiments, we combined the datasets from both experiments and ran mixed effects analyses that included Experiment as an additional factor in the models.

### RT results

Table 11 shows the findings of the linear mixed effects analyses conducted on the RT data from both experiments together. In the analysis of the mixed block, a significant main effect of Experiment reflected that, overall, participants were faster in Experiment 1 compared to Experiment 2 (cf. Tables 3 and 8). An overall significant main effect of Language indicated the robustness of the reversed language dominance across experiments. The significant main effect of TrialType showed that, overall, switching between languages in the mixed block came at a cost compared to not switching languages. Critically, the significant interaction between TrialType and Experiment confirmed that, statistically, the switch costs were overall larger in Experiment 1 compared to Experiment 2.

**Table 11:** Outcome of the linear mixed effects models performed on the RT data from Experiments 1 and 2 combined. Model structure reflects the model fit by maximum likelihood as indicated by the *buildmer* package. Significant *p* values are indicated in boldface.


1. Mixed Block comparison				

RT Switch Cost Model: Reaction Time ~ TrialType*Experiment*Language + (1 + Language*TrialType | Subject) + (1 + Language*TrialType | Item)				

	Estimate	SE	*t* value	*p* value

Experiment	173.68	33.80	5.14	**1.37e** ^-05^

Language	–103.09	15.03	–6.86	**8.59e** ^-09^

TrialType	36.61	6.54	5.60	**2.00e** ^-06^

Experiment × TrialType	–44.54	12.13	–3.67	**.001**

Language × TrialType	34.90	11.81	2.96	**.004**

Experiment × Language	–9.31	25.02	–.37	.71

Experiment × Language × TrialType	16.36	23.19	.71	.48

**2. Mixing Cost analysis**				

RT Mixing Cost Model: Reaction Time ~ TrialType*Experiment*Language + (1 + Language*TrialType | Subject) + (1 + Language*TrialType | Item)				

	Estimate	SE	*t* value	*p* value

Experiment	206.09	31.96	6.45	**3.15e** ^-07^

Language	–99.00	14.23	–6.96	**3.77e** ^-09^

TrialType	–307.68	17.45	–17.64	**<2e** ^-16^

Experiment × TrialType	17.69	11.69	1.51	.13

Language × TrialType	42.74	17.98	2.38	**.02**

Experiment × Language	5.86	22.13	.27	.79

Experiment × Language × TrialType	46.82	23.34	2.01	**.049**


*Note*: RT Switch Cost Model: Language (Dutch = –0.5; English = 0.5), TrialType (Language Repeat = –0.5 and Language Switch = 0.5), and Experiment (Experiment 1 = –0.5; Experiment 2 = 0.5) were sum-coded. RT Mixing Cost Model: Language (Dutch = –0.5; English = 0.5), TrialType (Language Repeat = –0.5 and Single-Language = 0.5), and Experiment (Experiment 1 = –0.5; Experiment 2 = 0.5) were sum-coded.

The overall mixing costs analysis statistically confirmed the reversed language dominance (main effect Language) and the overall asymmetry in mixing costs (interaction effect Language by TrialType). The significant three-way interaction confirmed the presence of a Language by TrialType interaction in Experiment 2, but not in Experiment 1.

### Accuracy results

Table 12 presents the findings of the logistic mixed effects analyses conducted on the error rate data from both experiments. In the analysis of the mixed block, the significant main effect of Language indicated the robustness of the reversed language dominance across experiments, also with regards to the error rates. The significant main effect of TrialType showed that, overall, switching between languages in the mixed block came at a cost compared to not switching languages. Overall, the proportion of errors observed in the mixed block differed only numerically across Experiment 1 (*M* = .071) and Experiment 2 (*M* = .058). The interaction between Language and Experiment indicated that, with regards to the error rates, the reversed language dominance was statistically larger for Experiment 1 compared to Experiment 2.

**Table 12:** Outcome of the logistic mixed effects models performed on the error rate data from the two experiments combined. Significant *p* values are indicated in boldface.


**1. Mixed Block comparison**				

Accuracy Switch Cost Model: ErrorRate ~ 1 + Language*TrialType*Experiment + (1 + Language | Subject) + (1 + Language | Item)				

	Estimate	SE	*z* value	*p* value

Experiment	–.19	.17	–1.15	.25

Language	–.58	.12	–4.75	**2.03e** ^–06^

TrialType	.49	.06	8.65	**<2e** ^–16^

Experiment × TrialType	–.14	.11	–1.22	.22

Language × TrialType	.15	.11	1.35	.18

Experiment × Language	.43	.22	2.00	**.046**

Experiment × Language × TrialType	–.27	.23	–1.18	.24

**2. Mixing Cost analysis**				

Accuracy Mixing Cost Model: ErrorRate ~ 1 + Language*TrialType*Experiment + (1 + TrialType + Language | Subject) + (1 + Language | Item)				

	Estimate	SE	*z* value	*p* value

Experiment	–.00	.23	–.01	.99

Language	–.22	.16	–1.40	.16

TrialType	–.46	.10	–4.62	**3.81e** ^–06^

Experiment × TrialType	.27	.14	1.94	.053

Language × TrialType	1.09	.15	7.43	**1.07e** ^–13^

Experiment × Language	.37	.25	1.47	.14

Experiment × Language × TrialType	–.53	.28	–1.89	.06


*Note*: Accuracy Switch Cost Model: Language (Dutch = –0.5; English = 0.5), TrialType (Language Repeat = –0.5 and Language Switch = 0.5), and Experiment (Experiment 1 = –0.5; Experiment 2 = 0.5) were sum-coded. Accuracy Mixing Cost Model: Language (Dutch = –0.5; English = 0.5), TrialType (Language Repeat = –0.5 and Single-Language = 0.5), and Experiment (Experiment 1 = –0.5; Experiment 2 = 0.5) were sum-coded.

In the analysis of the mixing costs in the accuracy data from both experiments, a significant interaction between Language and TrialType was observed. This interaction confirmed the asymmetrical mixing costs (i.e., mixing costs present for L1, absent for L2) in the error rates observed in both experiments.

## Interim Discussion

In Experiment 2, a new group of unbalanced but relatively proficient Dutch-English bilingual participants was immersed in a rich virtual environment in which they acted as owners of a market stand informing Dutch and English monolingual customers about the prices of fruit and vegetables using full sentences. As in Experiment 1, their speed and accuracy of responding were recorded and analyzed. In stark contrast with the computer setup used in Experiment 1, the overall richness of the experimental environment in Experiment 2 rendered participants’ utterances communicatively relevant: they produced full sentences for life-size (virtual) addressees who were helped by the information conveyed by the participant. Arguably, this setup resembles everyday communication substantially more compared to computer screen paradigms that have individual participants produce words or sentences in an isolated experimental booth, in the absence of an addressee for the produced utterance.

Across the board, the two markers of proactive, sustained inhibition commonly observed in this bilingual population were replicated in Experiment 2. Indeed, we again observed a robust reversed language dominance throughout the experiment, while mixing languages in the mixed block came at a cost compared to not mixing languages in the single-language baseline blocks. In contrast with Experiment 1 and five earlier experiments on this bilingual population (Peeters & Dijkstra, 2018; Peeters, 2020), however, we observed *asymmetrical* rather than symmetrical switch costs in the RTs from Experiment 2, which were asymmetrical in a direction that may seem surprising. Specifically, switching into the L2 came at a cost in the RTs, while switching into the L1 did not, reversing the theoretically influential RT pattern observed in the seminal study by Meuter and Allport (1999). We will now discuss the theoretical consequences of these observations in a General Discussion.

## General Discussion

Perhaps the single most important determiner of what language(s) a bilingual will select for speaking is the language background of the person they speak with. Surprisingly, the large majority of experimental studies investigating bilingual language production and control have done so in lab environments in which bilinguals are instructed to produce speech in the absence of an addressee, and as a function of an artificial cue presented on a computer screen (e.g., Meuter & Allport, 1999). To what extent are theories of proactive and reactive inhibitory control that rely on the results of such single-participant computer paradigms actually relevant for visually rich and communicative situations outside the lab?

In two experiments, we had Dutch-English bilinguals produce sentences as a function of a cue. While that cue was the traditional arbitrary color cue presented on a computer screen in Experiment 1, it was a 3-D monolingual addressee in Experiment 2, eliciting a sentence from the bilingual participant that was relevant to that addressee, and visually embedded in a rich marketplace environment in which participants acted as the owner of a fruit and vegetable stand. We reasoned that if well-established markers of proactive (reversed language dominance, asymmetrical mixing costs) and reactive (switch costs) inhibitory control mechanisms would be observed in such an environment, they would likely play a role in supporting bilingual language production in their everyday lives outside the artificial lab setting as well.

In Experiment 1, we replicated the findings observed in five earlier experiments testing this Dutch-English bilingual population: symmetrical switch costs, reversed language dominance, and mixing costs that were asymmetrical in the error rates (Peeters, 2020; Peeters & Dijkstra, 2018). While the five earlier experiments relied on a bilingual picture naming paradigm eliciting single-word responses, in the present Experiment 1 participants produced full sentences. The observed pattern of results indicates that having these participants produce full sentences, rather than single words, does not drastically modify the observed result pattern (see Table 13). Indeed, markers of reactive, trial-by trial inhibition (i.e., switch costs) and proactive, sustained inhibition of the L1 (reversed language dominance, larger mixing costs for the L1 than the L2) were found. As such, Experiment 1 served as the perfect baseline for enhancing the visual richness and communicative relevance of the setup in Experiment 2.

**Table 13:** Numerical size of the RT switch cost in ms in L1 (L1 switch cost: L1 switch – L1 repeat), the RT switch cost in L2 (L2 switch cost: L2 switch – L2 repeat) and the RT reversed language dominance (RLD: L1 Dutch – L2 English) as observed in the mixed block in seven experiments on different samples from the same unbalanced Dutch-English bilingual population.


EXPERIMENT	L1 SWITCH COST	L2 SWITCH COST	RLD

Peeters and Dijkstra (2018)			

Experiment 1	72	81	98

Experiment 2	67	63	122

Experiment 3	65	72	90

Experiment 4	69	63	86

Peeters (2020)			

Experiment 1	51	30	108

The present study			

Experiment 1	44	70	83

Experiment 2	–10	42	89


In Experiment 2, we again observed a clear reversed language dominance and mixing costs that were overall larger for the L1 than for the L2. In line with the Adaptive Control Hypothesis (Green & Abutalebi, 2013), these two findings have repeatedly been taken to mean that unbalanced bilinguals are capable of inhibiting their dominant L1 in a sustained fashion throughout an experiment (e.g., Declerck et al., 2020; Declerck et al., 2021; Peeters & Dijkstra, 2018; Peeters, 2020). In dual-language contexts such as a mixed block in a cued language-switching experiment, unbalanced bilinguals will typically not expect any difficulties with regards to communicating in their dominant mother tongue. As they are typically less proficient in their L2, slightly inhibiting their stronger L1 may decrease interference from that L1 and thereby facilitate language production in the L2. The asymmetry in mixing costs (i.e., the relatively larger increase in RTs and/or errors for the L1 compared to the L2 in the comparison of the mixed block with the single-language blocks) confirms this idea: inhibiting the L1 is particularly helpful when the use of both languages is required in a given context (Green & Abutalebi, 2013).

Besides similarities in results across both experiments, we also observed one important difference. While Experiment 1 yielded the symmetrical switch costs we have seen time and again in this Dutch-English population (cf. Table 13), Experiment 2 showed an asymmetry in switch costs in the RTs, as switching into the L2 came at a cost while switching into the L1 did not. This asymmetry was caused by RTs on the L1 repeat trials in the mixed block slowing down compared to the result pattern observed in Experiment 1 (cf. Figure 12). Asymmetrical switch costs have long been an influential finding supporting the Inhibitory Control Model (Green, 1998; Meuter & Allport, 1999). Indeed, using a cued language-switching paradigm, Meuter and Allport (1999) observed that unbalanced bilinguals had more difficulties switching into the dominant L1 than into their non-dominant L2. This observation empirically confirmed the idea that inhibition of a language via task schemas supports context-appropriate language selection processes in the bilingual mind (Green, 1998). As a stronger language requires a larger dose of inhibition when it should not be used, overcoming that inhibition (whether through active effort or passive dissipation of previously applied inhibition) when switching back into that stronger language should take longer than switching back into a (less-dominant) language that did not require such strong suppression (Meuter & Allport, 1999). We note, however, that the asymmetry in switch costs observed in the present Experiment 2 was actually asymmetrical in the opposite direction, that is, it was larger for the L2 than for the L1.

Theoretically, this switch cost asymmetry, like the opposite asymmetry observed by Meuter and Allport (1999), is in fact fully in line with the Inhibitory Control Model (Green, 1998). In our study, the bilingual participants clearly reversed their language dominance, such that they responded more quickly and made fewer errors in their L2 compared to their L1. As such, during the experiment, the language they are typically less proficient in temporarily became their stronger and more dominant language. In this overall context in which they proactively inhibited their L1 in a sustained fashion, the reactive, trial-by-trial inhibition applied to their L1 Dutch will therefore have been smaller than the reactive trial-by-trial dose of inhibition applied to their (temporarily dominant) L2 English. As such, their context-appropriate language selection process must have been supported by an interplay between both reactive and proactive inhibitory control mechanisms (Ma et al., 2016). Because of the overall similarity in design and procedure between the two experiments, the difference in result pattern must be caused by the enhanced degree of communicative relevance of the produced utterances and the increase in visual richness of the overall environment in Experiment 2.

Larger switch costs for the L2 compared to the L1 (‘reversed asymmetrical switch costs’) have been observed in a handful of earlier studies (e.g., Bonfieni et al., 2019; Liu et al., 2019; Ma et al., 2016; Sánchez et al., 2022; Timmer et al., 2019; Zheng et al., 2020). For instance, in the study by Sánchez and colleagues (2022), unbalanced Spanish-English bilinguals produced sentences in both a voluntary and a cued language-switching paradigm that used a network description task (cf. Declerck et al., 2021). In the cued condition, bilinguals used their L1 Spanish or their L2 English as a function of an artificial color cue, while in the voluntary condition for each trial they selected a language voluntarily. Whereas in the cued condition substantial switch costs were observed for both languages, the voluntary condition resulted in switch costs for the L2 but not for the L1. As in the present study, the more naturalistic setup (their voluntary condition; our Experiment 2) led to the switch cost asymmetry, while the less naturalistic setup (their cued condition; our Experiment 1) yielded statistically symmetrical switch costs. More generally, arguably more naturalistic experimental conditions, such as when bilinguals are allowed to use both languages they master and switch between these languages spontaneously, have sometimes shown a reduction in switch costs or even cost-free switching (Blanco-Elorrieta & Pylkkänen, 2017; De Bruin et al., 2018; Gollan & Ferreira, 2009; Gollan et al., 2014; Gross & Kaushanskaya, 2015; Jevtović et al., 2020; Kleinman & Gollan, 2016; Reverberi et al., 2018). The present study adds to this body of work the finding that also the presence of a natural language cue, such as a monolingual interlocutor, may make the switch cost disappear, at least for the bilingual’s second language.

The difference in findings between our two experiments must be explained by the degree of communicative relevance of participants’ utterances across the different experimental environments. In the current Experiment 2, participants addressed a life-size interlocutor that looked at them and expected a helpful response in a given language. In such an environment where there is something at stake, it is likely more important to produce a fluent, correct, and context-appropriate spoken utterance compared to a situation in which no addressee for one’s utterance is present at all, as in our Experiment 1. To prevent difficulties and disfluencies in speaking in their less dominant L2 English, our unbalanced bilingual participants may have applied a higher dose of sustained inhibition to their L1 Dutch in the more naturalistic environment, leading to relatively slow response times in their L1 across both repeat and switch trials (cf. Figure 12). As such, an increase in the communicative richness of one’s experimental set-up may lead to an increase of proactive and a decrease of reactive inhibitory mechanisms supporting bilingual language control. We deem it unlikely that our findings can be explained by enhanced relative activation of one language over another, as response times on L1 Dutch repeat trials became relatively slower in Experiment 2 compared to Experiment 1, rather than L2 English trials showing a relative decrease in RTs.

The present study also adds to a handful of earlier studies that had bilingual participants switch between sentences rather than between single words (Declerck & Philipp, 2015; Gollan & Goldrick, 2016; 2018; Gullifer et al., 2013; Johns & Steuck, 2021; Tarlowski et al., 2013). Whether switch costs are found in such studies seems highly dependent on the similarity between the languages under investigation (e.g., the degree of overlap in grammatical structures across languages) and the specifics of the task at hand (e.g., voluntary/spontaneous vs. cued vs. alternated language switches). We note that the Dutch and English sentences our bilinguals produced were highly similar in their grammatical makeup. In addition, though the variable words in the overall sentence structure were non-cognates (e.g., English *garlic*, Dutch *knoflook*), other words in the sentence were non-identical cognates (e.g., English *costs*, Dutch *kost*; English *cents*, Dutch *cent*). The overall grammatical and lexical similarity between the two languages in the produced utterances may have enhanced the need for effective application of inhibitory control more than for languages that would show significantly less lexical and grammatical overlap. In future work we will test the extent to which the present findings generalize to bilinguals mastering a more distinct language pair such as Mandarin Chinese and English.

As noted above, the main difference between the two experiments reported here lies in the communicative relevance of the participant’s behavior as induced by the broader visual context the experiment took place in. Our observation that *communicative context* modulates response patterns is in line with a wide variety of experimental studies in the field of cognitive science reporting differences in participant behavior as a function of their communicative intentions (cf. Becchio et al., 2012). For instance, participants modulate the velocity of their reach-to-grasp movement depending on whether they grasp an object for communicative versus non-communicative reasons (Sartori et al., 2009), and extend the duration of their pointing gestures if these are more informative for their addressee’s task (Peeters et al., 2015). As such, across different domains within the study of human cognition it is concluded that truly advancing our understanding of the cognitive mechanisms that support human communication may require at a very minimum the mere presence of an addressee in one’s experimental set-ups.

Methodologically, the present study confirms that the use of immersive virtual reality has great potential for studying the cognitive mechanisms that support language processing in this vein (cf. Huizeling et al., 2022; Misersky et al., 2022; Raghavan et al., 2023). Psycholinguistic studies of the mental processes that support language production in adults have for decades strongly relied on picture naming paradigms. While the use of confederates may provide the language user with an addressee in the lab, the use of confederates can be methodologically risky (Kuhlen & Brennan, 2013). By combining ecological validity with experimental control, and allowing for interaction with virtual agents that produce the exact same behavior with every new participant, virtual reality may provide the language researcher with a methodological basis for reliably studying how the cognitive architecture supporting (bilingual) language production and comprehension functions in context (Titus et al., 2024).

In sum, we conclude that theoretical models of bilingual language control must explicitly take into account the linguistic, visual, and interactive context in which bilinguals switch between languages if they wish to explain cognitive mechanisms involved in everyday bilingual communication. Monolingual interlocutors are natural language cues that may induce language selection and control processes in the bilingual mind and brain differently compared to artificial color cues presented on a computer screen. By taking the richness of the everyday environments in which our linguistic behavior typically occurs into the lab, our theories of cognition will more likely start to generalize to situations of natural human behavior (Blanco-Elorrieta & Pylkkänen, 2018; Dijkstra & Peeters, 2023; Hamilton & Huth, 2018; Hari et al., 2015; Hasson et al., 2018; Peeters, 2019; Willems, 2015).

## Data Availability

Raw data, analysis scripts, laboratory log, and a full pre-registration of the present study can be found on the OSF via: https://osf.io/5qp8x/?view_only=9ebc812967fc4d5abf0ce66147eee315.

## Footnotes

We originally pre-registered to exclude participants who obtained a score of 60 or lower on the LexTALE test. However, as we experienced difficulties in finding a large group of participants, we changed this lower limit to a score of 48. Note that the average LexTALE score across participants was nevertheless similar to that in previous studies testing samples from this bilingual population (cf. Table 1).

## Appendix A

Images of the sixteen target items (top four rows) and four practice items (bottom row) used as pictures in Experiment 1 and as 3-D objects in Experiment 2. Item names are presented in Dutch and English below the Figure.

Target item names in English: *Potato, Corn, Leak, Garlic, Eggplant, Pickle, Cauliflower, Peach, Pepper, Pineapple, Cabbage, Zucchini, Carrot, Onion, Orange, Lettuce*.

Practice item names in English: *Banana, Tomato, Pumpkin, Apple*.

Target item names in Dutch: *Aardappel, Mais, Prei, Knoflook, Aubergine, Augurk, Bloemkool, Perzik, Paprika, Ananas, Kool, Courgette, Wortel, Ui, Sinaasappel, Sla*.

Practice item names in Dutch: *Banaan, Tomaat, Pompoen, Appel*.

## Appendix B

Two-dimensional screenshots of the four virtual agents used as lifesize 3-D market stand visitors in Experiment 2.
